# Supplementary material for: A new plesiosaurian from the Jurassic–Cretaceous transitional interval of the Slottsmøya Member (Volgian), with insights into the cranial anatomy of cryptoclidids using computed tomography
Source: PeerJ. 2020 Mar 31;8:e8652. doi: 10.7717/peerj.8652 (PMC7120097; doi:10.7717/peerj.8652)
Supplement: Supplemental Information 1 [file peerj-08-8652-s001.docx]

# Supplementary Information 1 – Phylogenetic characters

The (1-270) characters utilised in this paper, are the characters complied and published in Benson and Druckenmiller (2014), with an addition of three new characters (271-273). For Characters (1-270), see the supplementary information for Benson and Druckenmiller (2014) available on Dryad for the character states and their history of use.

**271 Frontal, interfrontal vacuity (new character)**: frontals are loosely connected along the midline (0); frontals are partially separated along the midline by an interfrontal vacuity (1). Absent, frontals split entirely or partially by posterior process of the premaxilla or completely fused (?)

State (0) is present when the frontals are loosely sutured, but lack any emargination along the medial margin of the elements (e. g. *Muraenosaurus leedsii*; Figure S.11 A)

State (1) is only present in some cryptoclidid plesiosaurs (separate from the frontal foramen observed in some polycotylids) and *Brancasaurus brancai* (Sachs et al., 2016). When present, this state appears to be independent of ontogeny, as it has been confirmed in juvenile (e. g. NHMUK R2853) and adult specimens (e. g. *Tricleidus seeleyi*; Figure S.11 B).

Scored (0&1) in *Cryptoclidus eurymerus* and *Tatenectes laramiensis*, as a loosely sutured frontal is present, but the presence of a vacuity cannot be confirmed due to the preservation of the specimens.

**272 Dentary, mediolateral expansion of the dorsal surface (new character):**

No mediolateral expansion (0); a small lateral expansion present posteriorly; (1) mediolateral expansion, so the alveoli are laterally offset from the centre (2).

State (0) the dorsal surface of the dentary medial and lateral surfaces are uniform or convex and there is no or limited mediolateral expansion of the dentary in dorsal/ventral views (e. g. *Tricleidus seeleyi*; Figure S.12 A-B).

State (1): Some taxa show a lateral expansion of the dentary dorsal surface, although this is constrained to the posterior region of the dentary (e. g. *Cryptoclidus eurymerus*; Figure S.12 C).

State (2): some taxa show a mediolaterally extended dorsal surface of the dentary. This expansion is rapidly reduced on the medial and lateral surfaces, giving a triangular cross section at the midpoint of the dentary (e. g. PMO 224.248; Figure S.12 D-F).

**273 Morphology of the fibula (new character)**: Lunate, close to or as proximodistally long as wide (0); pentagonal anteroposteriorly wider than long, with equally sized distal facets for fibulare and astragalus (1)

Edited from character 92 in Smith (2007).

It should be noted that although the epipodials from the fore- and hind limbs in most plesiosaurs are similar in morphology, there are some differences in Cryptoclidid and Xenopsarian taxa.

State (0) is observed in most Early – Middle Jurassic plesiosaurians and pliosaurids (e. g. *Hauffiosaurus zanoi*; Figure S.13 A).

State (1) is observed in Cryptoclidid taxa (e. g. *Colymbosaurus svalbardensis*; Figure S.13 B)

**The impact of the new characters on the tree topology**

***Interfrontal vacuity***

Through examination of cryptoclidid specimens where the dorsal/ventral surface of the frontal is visible, an interfrontal vacuity along the frontal midline is clearly present in several taxa. In *Tricleidus seeleyi* (Fig. S.11B) and *Kimmerosaurus langhami* the medial margin of the frontal is slightly concave in dorsal view and has finished bone along the entire surface indicating the presence the interfrontal vacuity (AJR *pers. obs*.; NHMUK R3539; NHMUK R.8431). In *Cryptoclidus* *eurymerus*, the presence of this feature is ambiguous on the neotype (NHMUK R2860). In a referred specimen (PETMG R.283.412; Brown and Cruickshank, 1994), the elements are loosely sutured and a small vacuity is possibily present. Due to this *C. eurymerus* is scored for states 0 and 1 in the matrix. A similar situation is present in *Tatenectes laramiensis* (UW 24215 being poorly preserved, the medial surface of the frontal in a specimen of *Tatenectes laramiensis* (UW 24215) is smooth, indicating that a vacuity could be present (O’Keefe and Wahl, 2003a). This presence of this feature may not be limited to cryptoclidids; as a similar structure is observed in some xenopsarians, such as the Berriasian taxon *Brancasaurus brancai*, which preserves a small dorsomedian foramen along the frontal midline anterior to the pineal foramen (Sachs et al., 2016). Due to the uncertain occurrence in *T. laramiensis* and *Cryptoclidus*, and questionable homology in *Brancasaurus*, this character was not recovered as a synapomorphy for Cryptoclididae or any subclade. Additional cranial material in a broader sample of cryptoclidids is required to better understand the distribution of this character and its potential utility as a synapomorphy for this clade.

***Mediolateral expansion of the dentary***

A difference in the morphology of the dorsal (tooth bearing) surface of the dentary is observed between some cryptoclidid taxa and other members of Plesiosauria. In Early Jurassic taxa (with the possible exception of *Plesiopterys*), pliosauroids and xenopsarians, there is no mediolateral expansion of the dorsal dentary surface. Rather, the most common condition in plesiosaurians is that the lateral and medial surfaces are uniform and the alveoli are centred over the mandible. When present, a mediolateral expansion gives the mandible an inverted subtriangular shape in cross section, with the dorsal margin being widest. This feature is present in the majority of non-colymbosaurine cryptoclidids (with the exception of *Tricleidus seeleyi*), although in some taxa (*Cryptoclidus*), a partial lateral expansion of the dentary is only present posteriorly, or ambiguous between referred specimens possibly due to preservation (*Kimmerosaurus langhami*). The mediolateral expansion of the dentary unites *Spitrasaurus* spp. And *Ophthalmothule* on the strict consensus tree, however this character is poorly preserved in other taxa. This feature appears to have multiple states present in Cryptoclidids, with the ancestral state present in *Tricleidus seeleyi.*

***Fibula morphology***

In previous work, this character has been combined with the character describing radius morphology due to their morphological similarity (Smith, 2007, character 92). However, these are not homologous and should not be combined and differences between the ulna and fibula are evident in some cryptoclidid taxa (e.g. *Colymbosaurus svalbardensis*). In the resulting consensus tree, state (1) of this character was recovered as a synapomorphy for Cryptoclidia (Cryptoclididae + Xenopsaria).

**Data matrix**

The data matrix includes 76 OTUs and 273 characters. Missing data = “?”, dual character states = “()”. For the new characters (271-273): the character states for most OTUs were scored from the available literature, with the exception of the cryptoclidid taxa. These were scored from the literature in combination with personal observations on type and referred material (Supplementary Information 2).

**Time-calibration**

**Table S1.1:**

**Table over the first and last occurrences of taxa.**

Data from PBDB with some geological age mistakes corrected. Additional added occurrence for *Colymbosaurus svalbardensis* added from pers. obs (AJR) from MGUH collections.

|  | FAD | LAD | area |
| --- | --- | --- | --- |
| Plesiosaurus_dolichodeirus | 201.3 | 183 | 1 |
| Eretmosaurus_rugosus | 199.3 | 190.8 | 1 |
| Westphaliasaurus_simonsensii | 190.8 | 182.7 | 1 |
| Seelyosaurus_guilelmiimperatori | 183 | 182 | 1 |
| Microcleidus_tournemirensis | 180.2 | 175.6 | 1 |
| Microcleidus_brachypterygius | 183 | 182 | 1 |
| Microcleidus_homalospondylus | 182 | 175.6 | 1 |
| Plesiopterys_wildi | 183 | 182 | 1 |
| Cryptoclidus_eurymerus | 166.1 | 163.5 | 1 |
| Kimmerosaurus_langhami | 152.1 | 145 | 1 |
| Tatenectes_laramiensis | 163.5 | 157.3 | 3 |
| Djupedalia_engeri | 152.1 | 145 | 2 |
| Spitrasaurus_spp | 152.1 | 145 | 2 |
| PMO_224_248 | 152.1 | 145 | 2 |
| Tricleidus_seeleyi | 166.1 | 163.5 | 1 |
| Muraenosaurus_leedsii | 166.1 | 163.5 | 1 |
| Picrocleidus_beloclis | 166.1 | 163.5 | 1 |
| Pantosaurus_striatus | 163.5 | 157.3 | 3 |
| Plesiosaurus_mansellii | 152.1 | 145 | 1 |
| Abyssosaurus_nataliae | 132.9 | 129.4 | 6 |
| Colymbosaurus_megadeirus | 157.3 | 145 | 1 |
| Colymbosaurus_svalbardensis | 152.1 | 145 | 2 |
| Umoonasaurus_demoscyllus | 125 | 100.5 | 5 |
| Nichollssaura_borealis | 113 | 100.5 | 3 |
| Leptocleidus_capensis | 139.8 | 132.9 | 7 |
| Leptocleidus_superstes | 129.4 | 125 | 1 |
| MIWG_1997_302 | 129.4 | 125 | 1 |
| Cimoliasaurus_valdensis | 139.8 | 132.9 | 1 |
| Brancasaurus_brancai | 145 | 139.8 | 1 |
| Edgarosaurus_muddi | 113 | 100.5 | 3 |
| Plesiopleurodon_wellesi | 100.5 | 93.9 | 3 |
| QM_F51291_2 | 113 | 100.5 | 5 |
| GWWU_A3_B2 | 145 | 139.8 | 1 |
| Speeton_Clay_plesiosaurian | 132.9 | 129.4 | 1 |
| Wapuskanectes_betsynichollsae | 113 | 100.5 | 3 |
| Callawayasaurus_colombiensis | 125 | 113 | 4 |
| Futabasaurus_suzukii | 85.8 | 84.9 | 6 |
| Kaiwhekea_katiki | 84.9 | 66 | 5 |
| Aristonectes_parvidens | 72.1 | 66 | 4 |
| Libonectes_morgani | 93.9 | 89.8 | 3 |
| Hydrotherosaurus_alexandrae | 72.1 | 66 | 3 |

**Script used in R for time calibration**

**#** packages required *phytools*, *strap*, *paleotree*, *geiger*

#script for reading tree with singleton nodes. use read.newick instead

#nice feature to check that the range data and the tree data fit: check.names(tree, timedata) using geiger package

pt<-read.newick(file="filename.newick",)

pr<-read.csv("filename.csv", header=T, row.names = 1)

plio<-read.csv("filename.csv", header=T, row.names=1)

pt.ts <- DatePhylo(pt, pr, method="equal", rlen=5)

geoscalePhylo(tree=pt.ts, ages=pr, ranges=TRUE, cex.tip=1.5, cex.ts=2, cex.age=2, x.lim=c(40,210), quat.rm=TRUE, width=4, label.offset=1)

write.tree (pt.ts, file = "filename.newick")

**List of references for individual OTUs**

**Table A5.1:** List over references for the operational taxonomic units for adding the character states for new characters.

| **Operational taxonomic unit** | **References** |
| --- | --- |
| *Yunguisaurus liae* | Cheng et al., 2006; Sato et al. 2010; Shang et al., *in press*. |
| *Pistosaurus* postcranium | von Huene, 1948; Sues, 1987 |
| *Augustasaurus hagdorni* | Rieppel et al., 2002; Sander et al., 1997 |
| *Bobosaurus forojuliensis* | Dalla Vecchia, 2006; Fabbri et al., 2014 |
| *Anningsaura lymense* | Vincent and Benson, 2012 |
| *Stratesaurus taylori* | Benson et al., 2012, 2015 |
| *Avalonectes arturi* | Benson et al., 2012 |
| *Meyerasaurus victor* | Smith and Vincent, 2010 |
| *Maresaurus coccai* | Gasparini, 1997 |
| *Borealnectes russelli* | Sato and Wu, 2008 |
| *Rhomaleosaurus megacephalus* | Cruickshank, 1994; Smith, 2007, 2015 |
| *Archaeonectes* | NA |
| *Rhomaleosaurus cramptoni* | Smith, 2007; Smith and Dyke, 2008 |
| *Rhomaleosaurus zetlandicus* | Smith, 2007, 2013; Taylor, 1992a 1992b |
| *Rhomaleosaurus thortoni* | Smith and Benson, 2014 |
| *Thalassiodrracon hawkinsii* | Benson et al., 2011a; Storrs and Taylor, 1996 |
| *Hauffiosaurus longirostris* | Benson et al., 2011b |
| *Hauffiosaurus tomistomimus* | Benson et al., 2011b |
| *Hauffiosaurus zanoni* | Vincent, 2011 |
| *Marmornectes andrewi* | NA |
| *Peloneustes phiarchus* | Ketchum, 2008; Ketchum et al., 2011 |
| *Simolestes vorax* | NA |
| Pliosaurus BRSMGCs332 | NA |
| *Pliosaurus brachydeirus* | Knutsen, 2012 |
| *Gallardosaurus iturraldei* | Gasparini, 2009 |
| *Liopleurodon rossicus* | Halstead, 1971 |
| *Pliosaurus andrewsi* | Knutsen, 2012; Tarlo, 1960 |
| *Liopleurodon ferox* | Barrientos-Lara et al., 2015; Noè et al., 2003 |
| *Kronosaurus* | Cruickshank et al. 1999; Kear et al., 2006a |
| *Brachauchenius eulerti* | Schumacher et al., 2013 |
| *Brachauchenius lucasi* | Albright et al., 2011; Everhart, 2007; Hampe, 2005 |
| *Brachauchenius* MNA V9433 | NA |
| QM F51291 | Buchy et al., 2006 |
| *Attenborosaurus conybeari* | Wyse Jackson, 2004 |
| *Plesiosaurus dolichodeirus* | Vincent and Taquet, 2010 |
| *Eoplesiosaurus antiquior* | Benson et al., 2012 |
| *Eretmosaurus rugosus* | Brown, 1994 |
| *Westphaliasaurus simonsensii* | Schwermann and Sander, 2011 |
| *Seeleyosaurus guilelmiimperatoris* | Großmann, 2007 |
| *Microcleidus tournemirensis* | Bardet et al., 1999 |
| *Microcleidus brachypterygius* | Benson et al., 2012 |
| *Microcleidus homospondlyus* | Brown et al., 2013 |
| *Plesiopterys wildi* | O'Keefe, 2004 |
| *Cryptoclidus eurymerus* | Andrews, 1910; Brown, 1981; Brown et al., 1994 |
| *Muraenosaurus leedsii* | Andrews, 1910; Brown, 1981 |
| *Tricleidus seeleyi* | Andrews, 1910; Brown, 1981 |
| *Picrocleidus' beloclis* | Andrews, 1910; Brown, 1981 |
| *Tatenectes laramiensis* | O'Keefe et al., 2011; O'Keefe and Street, 2009 |
| *Pantosaurus striatus* | O'Keefe and Wahl, 2003b |
| *Plesiosaurus' mansellii* | Hulke, 1970 |
| *Spitrasaurus spp.* | Knutsen et al., 2012a |
| *Djupedalia engeri* | Knutsen et al., 2012b |
| *Colymbosaurus svalbardensis* | Knutsen et al., 2012c; Roberts et al., 2017 |
| *Colymbosaurus megadeirus* | Benson and Bowdler, 2014; Roberts et al., 2017 |
| *Abyssosaurus nataliae* | Berezin, 2011 |
| *Umoonasaurus demoscyllus* | Kear et al., 2006b |
| *Nichollssaura borealis* | Druckenmiller and Russel, 2008 |
| *Leptocleidus capensis* | NA |
| *Leptocleidus superstes* | Kear and Barrett, 2011 |
| *Cimoliasaurus valdensis* | Benson et al., 2013 |
| MIWG 1997 302 | NA |
| *Brancasaurus brancai* | Sachs et al., 2016 |
| GWWU As B2 | NA |
| Speeton Clay plesiosaurian | NA |
| *Wapuskanecttes betsynicholls* | Druckenmiller and Russell, 2006 |
| *Futabasaurus suzukii* | Sato et al., 2006 |
| *Callawaysaurus colombiensis* | Welles, 1962 |
| *Kaiwhekea katiki* | Cruickshank and Fordyce, 2002 |
| *Aristonectes paridens* | Gasparini et al., 2001, 2003 |
| *Libonectes morgani* | Sachs and Kear, 2017 |
| *Hydrotherosaurus alexandrae* | Welles, 1943 |
| *Edgarosaurus muddi* | Druckenmiller, 2002 |
| *Plesiopleurodon wellesi* | Carpenter, 1996 |
| QM F5191.2 | NA |

**Complete trees for Plesiosauria**

**References**

Albright III LB, Gillette DD, Titus AL. 2007. Plesiosaurs from the Upper Cretaceous (Cenomanian–Turonian) Tropic Shale of southern Utah, part 1: new records of the pliosaur *Brachauchenius lucasi. Journal of Vertebrate Paleontology* **27**:31-40.

Andrews CW. 1910. *A descriptive catalogue of the Marine Reptiles of The Oxford Clay Based on the Leeds Collection in the British Museum (Natural History).* British Museum (Natural History), London. 205 pp.+x plates

Bardet N, Godefroit P, Sciau J. 1999. A new elasmosaurid plesiosaur from the Lower Jurassic of Southern France. Palaeontology **42**: 927-952.

Barrientos-Lara JI, Fernández MS Alvarado-Ortega J. 2015, Kimmeridgian pliosaurids (Sauropterygia, Plesiosauria) from Tlaxiaco, Oaxaca, southern Mexico:*Revista Mexicana de Ciencias Geológicas* **32**:293-304.

Benson RBJ, Bates, K. T., Johnson, M. R. and P. J. Withers. 2011a. Cranial anatomy of Thalassiodracon hawkinsii (Reptilia, Plesiosauria) from the Early Jurassic of Somerset, United Kingdom. *Journal of Vertebrate Paleontology* **31**:562-574.

Benson RBJ, Ketchum HF, Noè LF, Gómez-Pérez M. 2011b. New information on *Hauffiosaurus* (Reptilia, Plesiosauria) based on a new species from the Alum Shale Member (lower Toarcian: Lower Jurassic) of Yorkshire, UK. *Palaeontology* **54**:547-571.

Benson RBJ, Evans M, Druckenmiller PS. 2012. High Diversity, Low Disparity and Small Body Size in Plesiosaurs (Reptilia, Sauropterygia) from the Triassic–Jurassic Boundary. *PLOS ONE* **7**:e31838.

Benson RBJ, Ketchum HF, Naish D, Turner LE. 2013. A new leptocleidid (Sauropterygia, Plesiosauria) from the Vectis Formation (Early Barremian–early Aptian; Early Cretaceous) of the Isle of Wight and the evolution of Leptocleididae, a controversial clade*. Journal of Systematic Palaeontology* **11**:233-250.

Benson RBJ, Bowdler T. 2014. Anatomy of Colymbosaurus megadeirus (Reptilia, Plesiosauria) from the Kimmeridge Clay Formation of the U.K., and high diversity amoung Late Jurassic plesiosauroids. *Journal of Vertebrate Paleontology* **34**:1053-1071

Benson RBJ, Druckenmiller PS. 2014. Faunal turnover of marine tetrapods of the Jurassic-Cretaceous transition*. Biological Reviews* **89**:1-23

Benson RBJ, Evans M and Taylor MA. 2015. The anatomy of *Stratesaurus* (Reptilia, Plesiosauria) from the lowermost Jurassic of Somerset, United Kingdom. *Journal of Vertebrate Paleontology* **35**:e933739.

Berezin, AY. 2011. A New Plesiosaur of the Family Aristonectidae from the Early Cretaceous of the Center of the Russian Platform. *Paleontological Journal* **45**: 648-660.

Brown, DS. 1981. The English Upper Jurassic Plesiosauridae (Reptilia) and a review of the phylogeny and classification of the Plesiosauia. *Bulletin of the British Museum (Natural History), Geology* **35**:253-347

Brown, DS. 1994. *Plesiosaurus rugos* Owen, 1840 (C’currently *Ertmosaurus rugosus*; Reptilia, Plesiosauria): proposed designation of a neotype. *Bulletin of Zoological Noemclature* **51**:247-249.

Brown, DS, Milner, AS and Taylor MA. 1986. New material of the plesiosaur *Kimmerosaurus langhami* Brown from the Kimmeridge clay of Dorset. *Bulletin of the British Museum (Natural History*) **40**:225-234

Brown DS, Cruickshank ARI. 1994. The skull of a Callovian plesiosaur *Cryptoclidus eurymerus*, and the sauropterygian cheek. *Palaeontology* **37**:941–953.

Brown DS, Vincent P, Bardet N. 2013. Oseological redescription of the skull of *Microcleidus homalospondylus* (Sauropterygia, Plesiosauria) from the Lower Jurassic of England. *Journal of Paleontology* **87**:537-549.

Buchy M-C, Frey E, Salisbury SW, Stinnesbeck W, López-Oliva JG, Götte M. 2006. *Neues Jahrbuch für Paläontologie Abhandlungen* **240**:241-270.

Carpenter K. 1996. A review of short-necked plesiosaurs from the Cretaceous of the western interior, North America. *Neues Jahrbuch für Geologie und Paläontologie. Abhandlungen* **201**:259p.

Cheng Y-N Sato T, Wu X-C, Li C. 2006. First complete pistosauroid from the Triassic of China. *Journal of Vertebrate Paleontology* **26**:501-504

Cruickshank ARI. 1994. Cranial anatomy of the Lower Jurassic pliosaur *Rhomaleosaurus megacephalus* (Stuchbury) (Reptilia: Plesiosauria). *Philosophical Transactions of the Royal Society of London B* **343**:247-260.

Cruickshank ARI, Fordyce RE, Long JA. 1999. Recent developments in Australian sauropterygians palaeontology (Reptilia: Sauropterygia). *Records of the Western Australian Museum* **1999**:201-205

Cruickshank ARI, Fordyce RE. 2002. A new marine reptile (Sauropterygia) from new Zeland: further evidence for a Late Cretaceous austral radiation of cryptoclidid plesiosaurs. *Palaeontology* **45**:557-575.

Dalla Vecchia FM. 2006. A new sauropterygian reptile with plesiosaurian affinity from the Late Triassic of Italy. *Rivista Italiana si Palenotologia e Stratigrafia* **112**:207-225.

Druckenmiller PS 2002. Osteology of a new plesiosaur from the Lower Cretaceous (Albian) Thermopolis Shale of Montana. *Journal of Vertebrate Paleontology* **22**:29-42.

Druckenmiller PS, Russell AP. 2006. A new elasmosaurid plesiosaur (Reptilia: Sauropterygia) from the Lower Cretaceous Clearwater Formation, North-eastern Alberta, Canada. *Paludicola* **5**:184-199.

Druckenmiller PS, Russell AP. 2008. Skeletal anatomy of an exceptionally complete specimen of a new genus of plesiosaur from the Early Cretaceous (Early Albian) of north-eastern Alberrta, Canada. Palaeontographica Abt. A, Paläozoologie-Stratigraphie **283**:1-33.

Everhart MJ. 2007. Historical note on the 1884 discovery of *Brachauchenius lucasi* (Plesiosauria; Pliosauridae) in Ottawa County, Kansas. *Transactions of the Kansas Academy of Science* **110**:255-258.

Fabbri M, Dalla Vechia FM, Cau A. 2014. New information on Bobosaurus forojuliensis (Reptilia: Sauropterygia): implications for plesiosaurian evolution. *Historical Biology* **26**:661-669.

Gasparini Z. 1997. A new pliosaur from the Bajocian of the Neuquen Basin, Argentina. *Palaeontology* **40**:135-147.

Gasparini Z. 2009. A new Oxfordian pliosaurid (Plesiosauria, Pliosauridae) in the Caribbean Seaway. *Palaeontology* **52**:661-669.

Gasparini Z, Casadio S, Fernandez M, Salgado L. Marine reptiles from the Late Cretaceous of northern Patagonia. *Journal of South American Earth Sciences* **14**:51-60.

Gasparini Z, Bardet N, Martin JE, Fernandez M. The elasmosaurid plesiosaur *Aristonectes* Cabrera from the latest Cretaceous of South America and Antarctica. *Journal of Vertebrate Paleontology* **23**:104-115.

Großmann F. 2007. The taxonomic and phylogenetic position of the plesiosauroidea from the Lower Jurassic Posidonia Shale of South-West Germany. *Palaeontology* **50**:545-564.

Halstead LB. 1971. *Liopleurodon rossicus* (Novozhilov) – A pliosaur from the Lower Volgian of the Moscow Basin. *Palaeontology* **14**:566-570.

Hampe O. 2005. Considerations on a *Brachauchenius* skeleton (Pliosauroidea) from the lower Paja Formation (late Barremian) of Villa de Leyva area (Colombia). *Mitt. Mus. Nat.kd. Berl., Geowiss. Reihe* **8**:37-51.

von Huene F. 1948. *Pistosaurus*, a Middle Triassic plesiosaur. *American Journal of Science* **246**:46-52.

Hulke JW. 1870. Note on some Plesiosaurian Remains obtained by J. C. Mansel Esq. F.G.S., in Kimmeridge Bay, Dorset. *Quarterly Journal of the Geological Society of London* **26**:611-622

Kear BP, Schroeder NI, Vickers-Rich P, Rich TH. 2006a. Early Cretaceous high latitude marine reptile assemblages from Southern Australia. *Paludicola* **5**:200-205.

Kear BP, Schroeder NI, Lee MSY. 2006. An archaic crested plesiosaur in opal from the Lower Cretaceous high-latitude deposits of Australia. *Biology Letters* : doi:10.1098/rsbl.2006.0504.

Kear BP, Barrett PM. 2011. Reassessment of the Lower Cretaceous (Barremian) pliosauroid *Leptocleidus superstes* Andrews, 1922 and other plesiosaur remains from the nonmarine Wealden succession of southern England. *Zoological Journal of the Linnean Society* **161**:663-691.

Ketchum HF. 2008. The anatomy, taxonomy and systematics of three British Middle Jurassic pliosaurs (Sauropterygia: Plesiosauria), and the phylogeny of Plesiosauria. PhD thesis. University of Cambridge, Cambridge.

Ketchum HF, Benson RBJ. 2011. The cranial anatomy and taxonomy of *Peloneustes philarchus* (Sauropterygia, Pliosauridae) from the Peterborough Member (Callovian, Middle Jurassic) of the United Kingdom. *Palaeontology* **54**:639-665.

Knutsen EM. 2012. A taxonomic revision of the genus *Pliosaurus* (Owen, 1841a) Owen, 1841b. *Noregian Journal of Geology* **92**:259-276.

Knutsen EM, Druckenmiller PS, Hurum JH. 2012a. Two new species of long-necked plesiosaurians (Reptilia-Sauropterygia) from the Upper Jurassic (Middle Volgian) Agardhfjellet Formation of central Spitsbergen. *Norwegian Journal of Geology* **92**:187-212

Knutsen EM, Druckenmiller PS, Hurum J. 2012b. A new plesiosaurid (Reptilia-Sauropterygia) from the Agardhfjellet Formation (Middle Volgian) of central Spitsbergen, Norway. Norwegian Journal of Geology **92**:213-234

Knutsen EM, Druckenmiller PS, Hurum JH. 2012c. Redescription and taxonomic clarfication of '*Tricleidus' svalbardensis* based on new material from the Agardhfjellet Formation (Middle Volgian). Norwegian Journal of Geology **92**:175-186.

Noè LF, Liston JJ, Evans M. 2003. The first relatively complete exoccipital-opisthotic from the braincase of the Callovian pliosaur, *Liopleurodon*. *Geological Magazine* **140**:479-486

O’Keefe FR. 2004. Preliminary description and phylogenetic position of a new plesiosaur (Reptilia: Sauropterygia) from the Toarchian of Holzmaden, Germany. *Journal of Paleontology* **78**:973-988.

O'Keefe FR, Wahl W. 2003a. Preliminary report on the osteology and relationships of a new aberrant cryptocleidoid plesiosaur from the Sundance Formation, Wyoming. *Paludicola* **4**:48–68.

O’Keefe FR, Wahl WJR. 2003b. Current taxonomic status of the plesiosaur *Pantosaurus striatus* from the Upper Jurassic Sundance Formation, Wyoming. *Paludicola* **4**:37-46.

O’Keefe FR, Street HP. 2009. Osteology of the cryptocleidoid plesiosaur *Tatenectes laramiensis*, with comments on the taxonomic status of the Cimoliasauridae. *Journal of Vertebrate Paleontology* **29**:48-57.

O’Keefe FR, Street HP, Wilhelm BC, Richards, CD, Zhu H. 2011. A new skeleton of the cryptoclidid plesiosaur *Tatenectes laramiensis* reveals a novel body shape among plesiosaurs. *Journal of Vertebrate Paleontology* **31**:330-339.

Rieppel OC, Sander PM, Storrs GW. 2002. The skull of the pistosaur *Augustasaurus* from the Middle Triassic of North-western Nevada. *Journal of Vertebrate Paleontology* **22**:577-592.

Roberts AJ, Druckenmiller PS, Delsett LL, Hurum JH. 2017. Osteology and relationships of Colymbosaurus Seeley, 1874, based on new material of C. svalbardensis from the Slottsmøya Member, Agardhfjellet Formation of central Spitsbergen. *Journal of Vertebrate Paleontology* **37**:e1278381

Sachs S, Hornug JJ, Kear BP. 2016. Reappraisal of Europe’s most complete Early Cretaceous plesiosaurian: *Brancasaurs brancai* Wegner, 1914 from the “Wealsen facies” of Germany. *PeerJ* **4**:e2813

Sachs S, Kear BP. 2017. Redescription of the elasmosaurid plesiosaurian *Libonectes atlasense* from the Upper Cretaceous of Morocco, *Cretaceous Research* **74**:205-222*.*

Sander PM, Rieppel OC, Bucher H. 1997. A new pistosaurid (Reptilia: Sauropterygia) from the Middle Triassic of Nevada and its implications for the origin of the plesiosaurs. *Journal of Vertebrate Paleontology* **17**:526-533.

Sato T, Hasegawa Y, Manabe M. A new elasmosaurid plesiosaur from the Upper Cretaceous of Fukushima, Japan. *Palaeontology* **49**:467-484.

Sato T, Wu X-C. 2008. A new Jurassic pliosaur from Melville Island, Canadian Arctic Archipelago. *Canadian Journal of Earth Sciences* **45**:303-320.

Sato T, Cheng Y-N, Wu X-C, Li C. 2010. Osteology of *Yunguisaurus* Cheng *et al.*, 2006 (Reptilia; Sauropterygia), a Triassic pistosauroid from China. *Paleontological Research* **14**:179-195.

Schumacher BA, Carpenter K, Everhart MJ. 2013. A new Cretaceous Pliosaurid (Reptilia, Plesiosauria) from the Carlile Shale (middle Turonian) of Russell County, Kansas, *Journal of Vertebrate Paleontology* **33**:613-628.

Schwermann L, Sander PM. Osteologie und Phylogenie von *Westphaliasaurus simonsensii*: Ein neuer Plesiosauride (Sauropterygia) aus dem Unteren Jura (Pliensbachium) con Sommersell (Kreis Höxter), Nordrhein-Westfalen, Deutschland. *Geologie und Paläontologie in Westfalen* **79**:5-60.

Shang Q-H, Sato T, Li C, Wu X-C. 2016. New osteological information from a ‘juvenile’ specimen of *Yunguisaurus* (Sauropterygia; Pistosauroidea). *Palaeoworld* **26:** 10.1016/j.palwor.2016.05.008.

Smith AS. 2007. Anatomy and Sytematic of the Rhomaleosauridae (Sauropterygia: Plesiosauria). PHD thesis. National University of Ireland, University College of Dublin. Dublin.

Smith AS. 2015. Reassessment of *‘Plesiosaurus’ megacephalus* (Sauropterygia: Plesiosauria) from the Triassic-Jurassic boundary, UK. *Palaeontologia Electronica* **18.1.20A**:1-19.

Smith AS, Dyke GJ. 2008. The skull of the giant predatory pliosaur *Rhomaleosaurus cramptomi*: implications for plesiosaur phylogenetics. *Naturwissenschaften* **95**:975-980.

Smith AS, Vincent P. 2010. A new genus of plesiosaur (Reptilia: Sauropterygia) from the Lower Jurassic of Holzmaden, Germany. *Palaeontology* **53**:1049-1063.

Smith AS, Benson RBJ. 2014. Osteology of *Rhomaleosaurus thorntoni* (Sauropterygia: Romaleosauridae) from the Lower Jurassic (Toarchian) of Northamptonshire, England. *Monograph of the Palaeontographical Society* **168**:1-40, pls 1-35.

Storrs GW, Taylor MA. 1996. Cranial anatomy of a new plesiosaur genus from the lowermost Lias (Rhaetian/Hettangian) of Street, Somerset, England. *Journal of Vertebrate Paleontology* **16**:403-420.

Sues HD. 1987. Postcranial skeleton of *Pistosaurus* and interrelationships of the Sauropterygia*. Zoological Journal of the Linnean Society* 90: 109-131.

Tarlo LB. 1960. A review of Upper Jurassic Pliosaurs. *Bulletin of the British Museum (Natural History), Geology* **4**:145-189.

Taylor MA. 1992a. Functional Anatomy of the Head of the Large Aquatic Predator Rhomaleosaurus zetlandicus (Plesiosauria, Reptilia) from the Toarcian (Lower Jurassic) of Yorkshire, England. *Philosophical Transactions: Biological Sciences* **335**:247-280.

Taylor MA. 1992b. Taxonomy and taphonomy of *Rhomaleosaurus zetlandicus* (Plesiosauria, Reptilia) from the Toarcian (Lower Jurassic) of the Yorkshire coast. *Proceedings of the Yorkshire Geological Society* **49**:49-55.

Vincent P. 2011. A re-examination of Hauffiosaurus zanoni, a pliosauroid from the Toarcian (Early Jurassic) of Germany. *Journal of Vertebrate Paleontology* **31**:340-351.

Vincent P, Benson RBJ. 2012. *Anningasaura*, a basal plesiosaurian (Reptilia, Plesiosauria) from the Lower Jurassic of Lyme Regis, United Kingdom. *Journal of Vertebrate Paleontology* **32**:1049-1063.

Vincent P, Taquet P. 2010. A plesiosaur specimen from the Lias of Lyme Regis: the second ever discovered plesiosaur by Mary Anning. *Geodiversitas* **32**:377-390.

Welles SP. 1943. Elasmosaurid plesiosaurs with a description of the new material from California and Colorado. *University of California Memoirs* **13**:125-254, pls.12-29.

Welles SP. 1962. A new species of elasmosaur from the Aptian of Colombia and a review of the Cretaceous plesiosaurs. University of California. *Publications of the Geological Society* **44**:1-96.

Wyse Jackson PN. 2004. Thomas Hawkins, Lord Cole, William Sollas and all: casts of Lower Jurassic marine reptiles in the Geological Museum, Trinity College, Dublin, Ireland. *The Geological Curator* **8**:11-18.
