# Supplementary material for: A new plesiosaurian from the Jurassic–Cretaceous transitional interval of the Slottsmøya Member (Volgian), with insights into the cranial anatomy of cryptoclidids using computed tomography
Source: PeerJ. 2020 Mar 31;8:e8652. doi: 10.7717/peerj.8652 (PMC7120097; doi:10.7717/peerj.8652)
Supplement: Supplemental Information 2 [file peerj-08-8652-s002.docx]

#NEXUS

begin data;

dimensions ntax=76 nchar=273;

format missing=? symbols="0~6";

matrix

Yunguisaurus_liae 0001000??10000000??0010010?000010010?000?000110??200??00?????01020???????????????02?10???????0?0?0010020???10011001100?1001?1????13??0??????1??????????41000??00?000000?001??0??01210?0?00{23}??0??????0?000?200????????????0?00122011?0000002000??11000?000?0?000000010000100000?0?

Pistosaurus_postcranium ????????????????????????????????????????????????????????????????????????????????????????????????????????????????????????????????????????????????????????20011100000000000110100101100010????101110?00?001?202100??0??001????0122210???1??????01?1?00000000???0???0?10????????0???

Augustasaurus_hagdorni 010100?0010?00000??00100100000000000000100?0010012101000010??????0???0?0000??????02??000?0110000?0010000????0?1100?10??1001??1???01010002100???00??21??41?01??000000200?0?10000011??0000???????????00?000?102?020000?0?????????????????000??00???100000000???0?100?1000???0?00?0?

Bobosaurus_forojuliensis ????????????????0??????????????????????????????????????????????????????????????????????????????????????????????????????????????????????02?0?00?00??01?2100000??110?12?000?00??001221100?10{23}1????????????????????????????????0122010?001??????0???1?000????????0???0?0??0?????????

Anningasaura_lymense 111000?0120?00??10000100?0010000100000?10000010112001000?10?0?1020?0110111010010?00211?1000000011?00?000?111??1110?100?1010?110??00010011000?0?00??0?0??10010??3?0?120100?031?1????????????????????????????????????????????????????????????????????????????????????????????????0?

Stratesaurus_taylori 001000?0100100100??0110010000010?0?000010000010001001000010101101012002000010?00102211???01000010110?000000?2?111?11000110001?0?001000011000?0?00??2001?10010011100120100003101??1????00?0????????????1?????????????????????01022111??????????????????????????1???010??010??10?00

Avalonnectes_arturi ??????????01??10????????10?????01??00001000001001100100001010???????????????????1??????0????????????????????????????????????????????????????{12}??????????2100?00211001??100?0110001101101200?0110?00????1?01201???????????????0102?110??1?????????11020?000?110110?101000010?100??0

Meyerasaurus_victor 10?00?0?21??00??11??0100??0000???0??????????????????10?0??0101??{01}0????????0??????022?0?1?01010111122?11000101?0111100?02110211???01?1001010?0???0???1?120?01?????00?20????0???0011211012002?110?0?0111?0?1????0201000?0001000102011?0011002100??12020?00021??11011010001101100?00

Maresaurus_coccai 110100?1220?1???1120{02}1001?0?00?????????????00001120?10???10?0110?0?????0?????????0??11?000101010?122?1000???200111?00??2100?11???01?1?011100????0???????0001???2?0?{01}??1???030??????????????????????????????????????????????????????????????????????????????????????????????????0?

Borealonectes_russelli 11010?1?210?102011100?00{12}0?0000??010?0010010000?{01}??1?000??0?0???20???12000???????022?1?0???????0??2???201?????111??00???1?0?110??0{12}?1?01?10?00100??21?120001???2?0??20?????30?0????????1???????????????????????????????????????????????100??00?????1101012???2?011?0001???11?1?0?

Rhomaleosaurus_megacephalus 100101212201121010002100100100?000?000010000010012001000110101???0?201?000???0??1002111000100011011???00?0002001111?0??20?0?11??0??00001020?0???0???10120001???1100?201????10?00111?1012001??20?0?0??????????1??????????????01022110????????00??1?0201001?110?1??1010??0?????0?00

Archaeonectrus_rostratus 1002002???0?102?1111??00?????????0?000?10000010012011000?1??01?????????????????????????????????????????????????11??1????1?????????????11120?0??????????2100100111000201???0?11?0110110110010??????0?????0?20?1??????????????01?2?1100?10001000??1?020?00??11011011010001101100???

Rhomaleosaurus_cramptoni 10000021220?1020100121101?00001?1000000???0001001201100011110?10?0???1?000???????02?1110?01010110122?1000000200?11?00??2100?11???01?1?01020?00?????????200???1211000??1?0?01?1?0002110?100?0????0?????1??????????????1??????0102?11000?10???00??1?021?001?1??110110100?11?1?00?00

Rhomaleosaurus_zetlandicus 11000??1220?102010012110??00001??000???????001001?0110?0?1110110?????1?0?0????????2211??0?1????1?1?2??0??0??2001111000021002110000??1001020?0??????????200010??110?020100?010?00??2110?1???01201000????????????????????????????????????1?0??001212021000121101101101001?????0??00

Rhomaleosaurus_thorntoni 11??0???2?0?10??1021?1101?00?????0??0001000?????????????1?????????????????????????????1??01??01?????????????200111??10?11???11000?11100101000???????????0001???110?000100??10??01?21101?00?0110100??01100120210?0?000100?10001220110001??????0111?02100002110??????????????????0?

Thalassiodracon_hawkinsii 00100001110100111120??0?1000???0000000?10020000001011000010110101010002000011000101011?00011001??100?000????0?11101100010000?1200?001001110010000??2101310010011100120100002100011101000001011000002011011001?00000001000?0001020111001000200000110100000010?11001010000101100?00

Hauffiosaurus_longirostris 00?20??0?1??12111120??00???2????????????????????02???????????01001??0???1?0?????1110?????????????110?0??0?????1001?100?1100?11?0023010011200?????????????????????????????????????????????????????????????????????????????????????????????????????????????????????????????????????

Hauffiosaurus_tomistomimus 00020?10210?1?110??0100?0?0210100100???????00?00010100?0?101?01011??????1001????111011?0?0100010?100?010??????1000?100?1100?112002301001120?1100???{02}?1231001?11110?100100002000011101010001??10???0???1??1?0?0????????????00????????00???0?1000012010000121?01101?1?0?11201?00?00

Hauffiosaurus_zanoni 00?2??1??1??????????????0?0??????????0?10???????????00?001???010????????????????11101101?0100010?100?010????20100??100?1100??12002301{01}01120000?00??????310011?11100100100?020?00?????????0?01100000201101120??0020000?010?0001020?2?0001001000??11010?00121??11011010001101100?00

Marmornectes_candrewi 0???????????00??0?????00???????????????????????0????10???0???0????????????????????????????????????????????????10100110?0000?111000201021020000000??21?1?0001???1200100100?200?1001??1?00?1?0010000??????1?0??000?00???00????00220111??2000??00110001000012100?1??1110??011???1???

Peloneustes_philarchus 10020120211100210??010001000101?010000010020010003121100010?00101000011000011?10102211100110{01}010?12110200???2110110110000001111000211001020001000??21?110001?121200110100000011001201100?1?0010001?00?1011000010000002010??1002301111022000?01??000210001220022101110111101111?00

Simolestes_vorax 110101???21?10210??010001??010??0100000100200?0003111?000?0?0010100??11000????1???221110???????0?121?0210???210111??10?0000?111000??1?01010?0??00???111100011121?0?1000001200110????11?????0????0??20?10012010?02?000?0????000230??1102201??010100021010122001110111001110???????

Pliosaurus_BRSMG_Cc332 110101?0221110210??0100110?0111?1100000100200100231111000201001010???1????01???0?012111001???????12110200???1?101?0010?200???13?00{12}11011011?????????????0001???1?00?00?????00???00??1100?????????????????????????????????????????????????????????????????????????????????????????

Pliosaurus_brachydeirus 11?1?????2??102?0??01001???0???????????????00?0???1??????????01010?????????????0?????????1?????0?12110200?????101??010?2000??13000{12}11101021?0???????????0001???110??10???0?00?2010??1100????????????????????????????????????????????????0???00????0?????1?1???3???1?0??10????1???

Gallardosaurus_iturraldei ??0?01?022?1??21????????10??????11?0000100200100?31?1100020?01101????1????????????121110???0?010?12110300???211?????1???0?????????????????1????00??2?11?00011??1?0?110?0??201??????????????????????????????????????????????????????????????????????????????????????????????????0?

Liopleurodon_rossicus 11?1?1??????10210??01000????1?1?11000001??200?0??3121??????????????????????????????????????????????????????????1????????0???????????1?01011???????????????????????????????????????????????????????????1000200?2????0?2???????????????????????????????????????????????????????????

Pliosaurus_andrewsi 01020?2021??10210??0??00??00???????????????00?00?3{01}211?0??0?00??10??????????????102?11?00110?010?1211020????21111?0110000101111000211001010001001102??210001012110010000002001100120110??1?0010000??????????????????????????00230110???200??01??02021010122003210?100011001211???

Liopleurodon_ferox 11010120221110210??010001000101?11?000010020000003121100020100101000?1100001??101022111001101110?12110210???2111110010?0010?111000111101010001100??0110100011121200100101000011000?0110????2010100????1??0?000?????0??01???1????????10320???010???02101012?0?2?101?0001???1?11???

Kronosaurus_MCZ_1285 ??0?0?20?2??????0?????????????????????????????????????????01000?{01}?00011000?01110?111?0???????????12110{23}10??????????0????001????0??????01010?011?0??2??00000?11?3211?0?000?0001??0?20?1??0??2?10000?00?10?0?????02??0020????1????????103??????????????????????????????????1???????

Brachauchenius_eulerti 010200?0211100210??1100010?0200?11100001012001002312100012??00???????????????????011?0?00011000101211031010?21110?0010?0001?11???00000010100???????????????????1???????????????????????????????????????????????????????????????????????????????????????????????????????????????0?

Brachauchenius_lucasi 00020120211100210??010001000200?111?0001012001000312100012??00???????????????????011?0100?1011?0??2100310???211???001?00001{12}11????????010?0?0???0?????000002????211?00?????????00??001???????????????????????????????????????????????????????????????????????????????????11111?0?

Brachauchenius_MNA_V9433 0102?????1?100210??0100010?0????????0001???00?002{23}1210?0?20?0????0?????????????????????00????110??2110210???2111000010?0001?1110000000010100???????????0000211?3211100000?200?2?0???01??????????????0?1???????22200002?????11??0011?10???????????????????????????????????????????

QM_F51291 ????01??2?????210??010?????0210?1110000????0010???12?????????????????????????????????0?00?????????????{23}??????????????????????????????????10??????????????????????????????????????????????????????????????????????????????????????????????????????????????????????????????????????

Attenborosaurus_conybeari 00010010???110??112???0010?0?????0??0001002??????2??10000???11??????????????????1?2???????????????????????????0110??0???0?????????10100???0?2??????????310??011110????1???0{03}00??11101100?0???1???1?20?10?1????0220000?000?310?02??1?0010001000??1??10?00?????1?001?100???01101?0?

Plesiosaurus_dolichodeirus 001100?0110?00100??0010010?000010010000100?001000100100001010110{01}010?02000?1111?10?01100?01000110100?000?1110?0100?100?1000???????100001110?2???0????01410010121{01}00100101003{01}00011111100001000010{01}?211101100110000000?000?000002012100100020000?1102010000200110{01}1010000?01100??0

Eopleiosaurus_antiquior ????????????????????????????????????????????????????????????????????????????????????????????????????????????????????????????????????????????2??????????4100?0111?0?12?1???03??0??????????01000?11?02??1001002?0?0??00???????0??2?11?00110020000?11010100??1?0110?1010000101100??0

Eretmosaurus_rugosus ????????????????????????????????????????????????????????????????????????????????????????????????????????????????????????????????????????????{12}??????????{34}100110?100?12010000?000111??11?0???0000?01????1???????{01}?0??0???????0010201200011000000??11021?100020011011010000201100???

Westphaliasaurus_simonsensii ????????????????????????????????????????????????????????????????????????????????????????????????????????????????????????????????????????????2????????????0011021?0??0010?1031000111?1?0010?0000101?211100120??00000001??10000??20?20001?????00011002100011100110110100011?1??1??0

Seelyosaurus_guilelmiimperatori 00000?00?10?02?????0???????0?????0??0011001????1?1?01000?0??11?????????????????????????????????????????????????10??100?1010???????100001100?2??????????3100?1221000???1???00?00?1200110010{23}100001101101001201?10030?1?0???300?02??200011000100??11020?100?20011011000011201101?0?

Microcleidus_tournemirensis 0000000?120102100??0010010000000001011110010010002001000110?1???{12}1?????000?????0112?110000??00?0?12100100???20????????????????????11000110012?????????142101112100?20?100?0010011200111010?????????201100120211013010?010?300122012000110????0??1?020?100?2?0110110100012?1??1??0

Microcleidus_brachypterygius 0011000?110102100??0010010000000101011110010010102001000100?????1???????????????101??100?0010000?12100000???20?????????????????????0000110012?????????2321011121?00??1????00100?12011110?03100011?0210???1????20130?0?01???101220?10??01002000??11021?100?2??11010010011101101???

Microcleidus_homalospondylus 0000000011010210???0010020?000?010?0111100100101020010001??11110110010200001??001021?1?00????000??2100100???2001001??00?0???102???10000??0012??????????42101112100020110010010011201111010?1000011??1011110001?01?010101???00122012000010?20000011021?100110011011010011201101?0?

Plesiopterys_wildi 00100?0?1???00????????00???00??1?0?0???10??01?0001001000?00?0?10101110200001001010110???1????0011?00??00?100???20??100?1000?0?2??0?00?01110??????????02420011121101?001??0001001111?1100003000000?01?11?01201??0??0???010?3?0102012?001???00?000110200101?2??11001010011101110010

Cryptoclidus_eurymerus 0010010010?101100??021001010001100?0020100201001011010000000010?1011101101?111011011??00101100011?00?0????000?02001100?1000?0021?0200001110121111101?013100111232011001110000000111011000000010001121011001010201201020200?1100210110112010?101113032010122101300110{12}2{01}{01}001211{01}11

Tricleidus_seeleyi 011000??????011010?00?0010?000?100????????{12}01001?3001000?000010?00?0111111?????110110?????????011200?000?1100?0201110001000?0021?01110011001?11?1101?02210011123201100111000100?11?01100???????????110110000002012010202?13????????????210??1011130210101221023001101111101211101

Muraenosaurus_leedsii 00????????0?011010?001001?0000110??????????010?0?????1???0???10?00?0????11??0??11011??????1???????0???????0???0201010002000?0021?010100110012?1?1101??14210112232012001110001001??11110000?200?111???????0???0{12}????1?2020?3?1012?021??12????1001230311101221023001100111001210121

Kimmerosaurus_langhami ??1??0?1??0?0????????????0?????10??0??????20100000102000000011101010201101?10111??11?????????????200?0????????0200110001000?002??0?00?01220???1?1001?00?00011??3?01100111???1?0???????????????????????????????????????????????????????????????????????????????????????????????1{12}?

Pantosaurus_striatus ???????????????????????????????????????????????????????????????????????????????????????????????????????????????????????????????????????????????????????{34}100111?320??00???000100??1??110000?2000111?????????????????????????01001?021011200??10????0210101??1?1?10???211???1211???

Picrocleidus_beloclis ?????????????????????????????????????????????????????0????0?010?{01}0?????????????10011??????????????????????????????1100?1000???2??2?????11?????1?1101?02411011223201200111000100111?011?0?0?200?101001011002010201201020201?01?12?021112200??101???02111012210110010011111?1?10?1?

Tatenectes_laramiensis ???????0?????????????????0??????0??????1????????????2000?0000??????????????????????????????????11????????0?1???????????????????????????11??????01001????00011??32011001?1000100?110?110000??????????1011??????{12}01??1020101?10?110021011210??10????0210101??1?2?001?0211??01??1{01}??

Plesiosaurus_mansellii ?????????????????????????????????????????????????????????????????????????????????????????????????????????????????????????????????????????????1111101??2{34}1101???320??00?????01?01??1111?????1010011??0???????????2??1?202????????????????????????????????????????????2???????????1

Colymbosaurus_megadeirus ???????????????????????????????????????????????????????????????????????????????????????????????????????????????????100?1000??????0???????????1111110??2410012??320?200111??01?01??1011??00?2110101??0???????????2??102?2???????????????200??10101?0210101221022101102?11101211??1

Djupedallia_engeri ??????????????????????????????????????????????????????????00???????0?01111????????????????????????????????????020??????????????????00?0??1??2011???100?510012113201200111030100110??1100??????????????10?0???0?0??01???{12}??3????????????2?0??????1?02?0?012?1???101102???1?1211??1

Spitrasaurus_spp ??????0???????????????????????????????????????????????0???00??1010?????????????1??????????????????0???????????0201?100?1000???2??0?00?01110?20111111??25210122?3201200111030100110?01100??????????????10?0???0?0??01???2???????????????2?11?????1?02{12}0?012?1???101102?1???1{12}11?2?

Abyssosaurus_nataliae ????????????????0??????????????????0???????0???????0?????????????????0?????????????????????????????????????????????10???????????????????????2???1110??1{45}1001???3201200111??01?01??1011??0??{12}0???????0010000100??2??10?0?001?1?1101110??2?1??1????10???10?22?0?3??110{02}??1001211??1

Umoonasaurus_demoscyllus 00000100112??1101020??00??00????0??????????0?002222?10???10?11????1??12?11?0??0??022?????0?????11122?1211000????????0??1111???????1111010?01????????????1001????20?1??1010??00?110101102000201?00?1???1?01?010?????????????0???????????21????0111?02001022210?2???1?0??10?1??1???

Nichollssaura_borealis 0000010011200010102010001100000100?000010010000122301010010011??211?2??0???00000?0221110101100011122?13110000?0111110??0101?112??02?1001010100100??20022100100132011?010??000??1102?101?00000?????????10110020??????????????2?12102?01?2?{01}0000??1??200101?21012101100011101110?11

Leptocleidus_capensis 010001?1112?1010102002001?0000?10??0000??01001022{23}201010?0??01??21?????0?????????0221110?01?00011122?12110001?????1110?0111??{12}2?111111010101???????????210010??320?1?0100?0?0?0?????1??????????????????????????????????????????????????2????00????0???????????2???1?0??10?1??????

Leptocleidus_superstes ??0?0??1?1?0????10{12}?????1??????????????????0?10222201010?100011121?0011?01???????02211???????????122??31????1??????????????????????????????????????????{12}0?01?0132011001000??00?110?11102???1011001?10110011020??1?100200102??????????????????01???02001012?0?????????????????????

Cimoliasaurus_valdensis ?????????????????????????????????????????????????????????????????????????????????????????????????????????????????????????????????????????????1100???1?1{12}1001002320?110100000001?????1??????1011000??????????????????????????????????????????001???02001021?0????????0????????1???

MIWG_1997_302 ??????????????????????????????????????????????????????????????????????????????????????????????????????????????????????????????????????????????????????????01?01??0?1??1010??00??10??1102?????????????1???1?02???1??01?10?0???????????????????????????????????????????????????????

Brancasaurus_brancai 0010?000100000{01}?101002001?00000?0?1000???0101?02223010?0?00?011101101120101000100022?????011000???0???????????01???100?1110???2??121100111?120100??20014100100232012?0101100000110101102000101?1011???1?01?0201?10?0?210?0201012102101121????0111002001022200321?11???110?1???1?0

GWWU_A3_B2 ??????????????????????????????????????????????????????????????????????????????????????????????????????????????????????????????????????????????????????????01?12?20?2001111?000?1110011100002011101????1????????????0?210???01012102101021????0112?02000022210?1???1?0????????1???

Speeton_Clay_plesiosaurian ????????????????????????????????????????????????????????????????????????????????????????????????????????????????????????????????????????????2???????????2101012320120011110010011201111000?20?1111??1011?1??20??1??002?????010121011011200??00102103001022?1021001100011101?11???

Wapuskanectes_betsynichollsae ?????????????????????????????????????????????????????????????????????????????????????????????????????????????????????????????????????????????????????????????????????????????????1??1??0???????????2101{01}2?110?1110110?10??3????????????200???01???03000022???????????????????0???

Futabasaurus_suzukii 00??000?1?0?00??0??00?0?2?0000????????????????????0???1??????????????????????????????????0110000???????????????100??????0????{12}????100001100???????????????11?22?2022001111?010?11110111000??????????????????????????????01301?1??01?0?1100200000220300102221122101100011101{12}10?01

Callawayasaurus_colombiensis 000010?0?10000{12}?10100?0020?000?10????????01010000220101010001111011?{12}02?1010??1?002211001????0?0???2?1100???1?1101{01}?0??20?????2??010000110012??????????5210102232022001111{01}0100111211110???????????200100?11001120010?10?01?????????00??0{01}??100?220300102?3101200?10{01}0111??2?1???

Kaiwhekea_katiki 00001010??000???1020020020?000?????????11????0?0?32?2111000?00????????????????????????????????????????????????0101?10??1000??????0300001120?2??????????51011122320?2?0111?10100?11111?10???2????0??????????????????????????????????????000??00??11?32?11??3?12110100{01}0012?1??0???

Aristonectes_parvidens 00?010??1?0000201??00200200000??00???{01}?11??01010?3212?1?0000?00?1?01212010???????????????0?10000??????????????0101010??1000???????3000011200?1101101?02?1111???3???200111??01?0????????????????????????????????????????????????????????????????????????????????????????????????0?

Libonectes_morgani 010010?011000020102002002000000?00110001001110??0?21??111?00010?010?20201010?00100221100101?00?0?122?1200???2101000101?2000?112000101001?0012???1?????1621?122?320?200111??01??1???????????????????21011??????11100?0?100?1?????????????00??00????????10??????200??01?1????????0?

Hydrotherosaurus_alexandrae 000010?0??0000201{12}100?002??0000?0011000100111?????2121?111??01?????????????????????????????????????????????????100010?1?01??112???10000110012???1?????1521112223202200111?101001110011100002011?11?1001{01}00001011200?020????120111011000100?000??12031010123102300110{01}011101211???

Edgarosaurus_muddi 100101?021001020111010001??0?0??001000010?200102220011101000110?01?02?2?1?0????1002211?01??????10122?1300100201100011011110?120011{23}110111100?010100000121001?1132011201000000000???01????????????????????????????????????????????????????00?00??????0010?2?0?3?10??0101??01211?0?

Plesiopleurodon_wellesi 000100?0?100102?1120?000???2?0?????????????1???222201010?10?11????????????????????????????????????????????????0001?11???110?1????1{12}000210?0?????????????100?011320?120100?0?0?0??0??110{02}???{01}0??101????????????20110?1?01???????????????????????????{23}{01}?1012?1?????????????????????

QM_F51291.2 00010020110011201120?000{12}??2?0??00??01010??1???2222010101100111101?????????????1?022??001???00???1?2?100????1??001??????1???11????100011020?11101000?0210001{01}02320?1201001000100100?1100?00101010011??1???????20?1??????????20100121011200110110111200101221022101100011101{12}10???

Colymbosaurus_svalbardensis ????????????????????????????????????????????????????????????????????????????????????????????????????????????????????????????????????????????????????????????????????????????????1???110000?21101011?0??????????0??????0??0??1?1101?1???200??1010120?101012{12}1023101102111101211??1

PMO_224.248 00100000210?0101102021001?1000110??????????010?102102??0000?110?1010?01?1???111100110?00?01000011200?000?110??02??010001000???2??0200001100120111111???52001{12}2?320120011103010011??0110????????????21011001010201?010202?13????????????201??100???02001022?1?2?101?0201???121112?

;

end;

begin trees ;

tree tnt_1 = [&U]

(Yunguisaurus_liae ,(Pistosaurus_postcranium ,(Augustasaurus_hagdorni ,(Bobosaurus_forojuliensis ,(Eopleiosaurus_antiquior ,(((Stratesaurus_taylori ,(Anningasaura_lymense ,Avalonnectes_arturi ,Rhomaleosaurus_megacephalus ,(Archaeonectrus_rostratus ,(Meyerasaurus_victor ,((Maresaurus_coccai ,Borealonectes_russelli ),(Rhomaleosaurus_thorntoni ,(Rhomaleosaurus_cramptoni ,Rhomaleosaurus_zetlandicus ))))))),(Plesiosaurus_dolichodeirus ,(Eretmosaurus_rugosus ,(Westphaliasaurus_simonsensii ,((Seelyosaurus_guilelmiimperatori ,(Microcleidus_tournemirensis ,(Microcleidus_brachypterygius ,Microcleidus_homalospondylus ))),(Plesiopterys_wildi ,(((((Cryptoclidus_eurymerus ,(Kimmerosaurus_langhami ,Tatenectes_laramiensis )),(Djupedallia_engeri ,(Spitrasaurus_spp ,PMO_224.248 ))),(Tricleidus_seeleyi ,(Muraenosaurus_leedsii ,Picrocleidus_beloclis ))),((Pantosaurus_striatus ,Plesiosaurus_mansellii ),(Abyssosaurus_nataliae ,(Colymbosaurus_megadeirus ,Colymbosaurus_svalbardensis )))),((((Umoonasaurus_demoscyllus ,Nichollssaura_borealis ,(Leptocleidus_capensis ,Leptocleidus_superstes )),(MIWG_1997_302 ,(Cimoliasaurus_valdensis ,Brancasaurus_brancai ))),(Edgarosaurus_muddi ,(Plesiopleurodon_wellesi ,QM_F51291.2 ))),(GWWU_A3_B2 ,(Speeton_Clay_plesiosaurian ,((Wapuskanectes_betsynichollsae ,Callawayasaurus_colombiensis ),((Futabasaurus_suzukii ,(Kaiwhekea_katiki ,Aristonectes_parvidens )),(Libonectes_morgani ,Hydrotherosaurus_alexandrae ))))))))))))),(Thalassiodracon_hawkinsii ,((Hauffiosaurus_longirostris ,Hauffiosaurus_tomistomimus ,Hauffiosaurus_zanoni ),(Attenborosaurus_conybeari ,(Marmornectes_candrewi ,(Peloneustes_philarchus ,(Pliosaurus_andrewsi ,(Simolestes_vorax ,(Liopleurodon_ferox ,((Liopleurodon_rossicus ,(Pliosaurus_BRSMG_Cc332 ,(Pliosaurus_brachydeirus ,Gallardosaurus_iturraldei ))),(Kronosaurus_MCZ_1285 ,Brachauchenius_eulerti ,Brachauchenius_lucasi ,Brachauchenius_MNA_V9433 ,QM_F51291 ))))))))))))))));

end ;
