# Supplementary material for: A new plesiosaurian from the Jurassic–Cretaceous transitional interval of the Slottsmøya Member (Volgian), with insights into the cranial anatomy of cryptoclidids using computed tomography
Source: PeerJ. 2020 Mar 31;8:e8652. doi: 10.7717/peerj.8652 (PMC7120097; doi:10.7717/peerj.8652)
Supplement: Supplemental Information 3 [file peerj-08-8652-s003.docx]

# **Supplementary Information 3**

# **Body length estimations**

Body length estimations for PMO 224.248 were calculated using the know length of the vertebral column and intervertebral spacing observed during preparation. Estimations of tail length if incomplete, was based on more complete specimens of comparable size. Body size estimates for *Cryptoclidus eurymerus* (HMG V1091) and *Muraneosaurus leedsii* (NHMUK R.2422) are already published in Brown (1981).

# **Mechanical advantage**

Mechanical advantage was calculated using the methodology outlined by Stubbs et al., (2013). For individual specimens with parts of the lower jaw missing, the outlever measurement was estimated using the size of the upper jaw and skull. Mechanical advantage is generally associated with weaker, but rapid biters, whereas high mechanical advantage is associated with a high bite force.

Two measurements of mechanical advantage were calculated for the specimens, Anterior mechanical advantage (AMA) and posterior mechanical advantage (PMA). The data from this is available in supplementary information table S.5.

**Figure S.2.1:**

**A diagram illustrating the lengths of measurements for the different levers.**


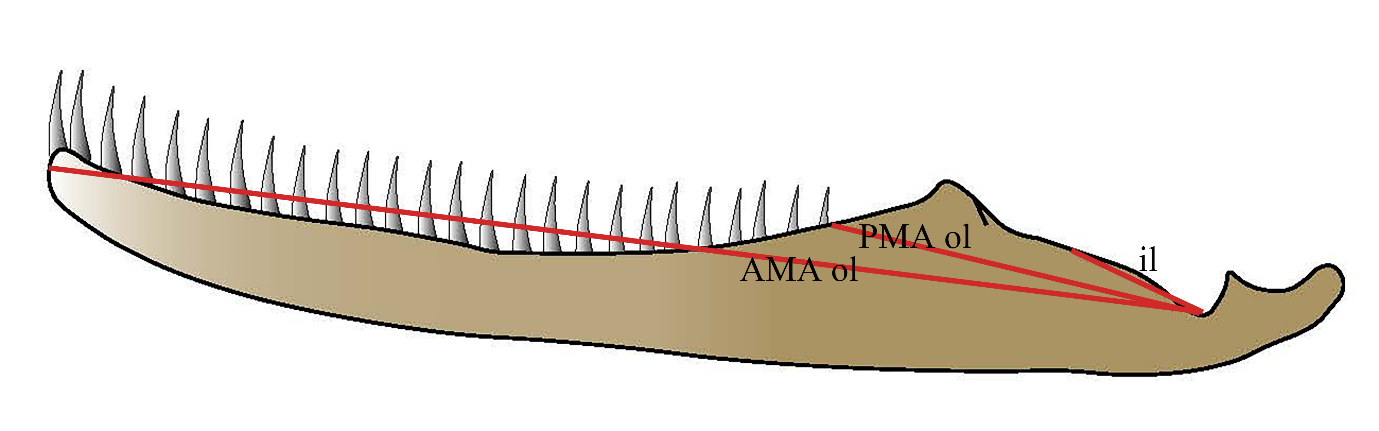


**References:**

Brown DS. 1981. The English Upper Jurassic Plesiosauridae (Reptilia) and a review of the phylogeny and classification of the Plesiosauia. Bulletin of the British Museum (Natural History), *Geology* **35**:253–347.

Stubbs, TL, Pierce, SE, Rayfield, EJ, Anderson PSL. 2013. Morphological and biomechanicaldispariy of crocodile-line archosaurs following the end-Triassic extinction. *Proceedings of the Royal Society* *B* **280**:20131940
