# Supplementary material for: A new plesiosaurian from the Jurassic–Cretaceous transitional interval of the Slottsmøya Member (Volgian), with insights into the cranial anatomy of cryptoclidids using computed tomography
Source: PeerJ. 2020 Mar 31;8:e8652. doi: 10.7717/peerj.8652 (PMC7120097; doi:10.7717/peerj.8652)
Supplement: Supplemental Information 4 — Measurements in millimetres, “~” indicates uncertainty due to damage. [file peerj-08-8652-s004.docx]

**Table S.1:**

**Selected cranial measurements from PMO 224.248 in mm.**

Measurements in millimetres, “~” indicates uncertainty due to damage.

| **Cranial measurements of PMO 224.248** |  | **mm** |
| --- | --- | --- |
| Anteroposterior length of skull |  | 225 |
| Mediolateral width of skull posteriorly |  | 112 |
| Mediolateral width of skull over maxilla (max) |  | 120 |
| Anteroposterior length of premaxilla |  | 82 |
| Anteroposterior length of frontal |  | 70 |
| Mediolateral width of frontal (max) |  | 39 |
| Anteroposterior length of parietal |  | 30 |
| Dorsoventral height of squamosal |  | ~41 |
| Anteroposterior length of orbit |  | 60 |
| Mediolateral width of orbit |  | 55 |
| Anteroposterior length of skull anterior to orbit |  | 86 |
| Anteroposterior length of pterygoid |  | 122 |
| Mediolateral width of left quadrate over condyle |  | 24 |
| Mediolateral width of basisphenoid posteriorly |  | 16 |
| Anteroposterior length of antorbital fenestra |  | 80 |
| Anteroposterior length of posterior interpterygoid |  | 3 |
| Mediolateral width of vomer |  | 29 |
| Anteroposterior length of left palatine |  | ~100 |
| Mediolateral width of basioccipital condyle |  | 21 |
| Dorsoventral height of basioccipital condyle |  | ~16 |
| Anteroposterior length (ventral) of basioccipital condyle |  | 26 |
| **Left mandible** |  |  |
| Anteroposterior length (preserved) |  | 175 |
| Anteroposterior length of retroarticular process |  | 34 |
| Medial-mesial width (max) of dentary |  | 20 |
| Mediolateral width of glenoid |  | 18 |
| Dorsoventral height |  | 31 |
| **Right mandible** |  |  |
| Anteroposterior length (preserved) |  | 140 |
| Anteroposterior length of retroarticular process |  | 33 |
| Medial-mesial width (max) of dentary |  | ~12 |
| Mediolateral width of glenoid |  | 18 |
| Dorsoventral height |  | 33 |
