# Supplementary material for: A new plesiosaurian from the Jurassic–Cretaceous transitional interval of the Slottsmøya Member (Volgian), with insights into the cranial anatomy of cryptoclidids using computed tomography
Source: PeerJ. 2020 Mar 31;8:e8652. doi: 10.7717/peerj.8652 (PMC7120097; doi:10.7717/peerj.8652)
Supplement: Supplemental Information 5 — Abbreviations: aa, atlas-axis complex; H/L, height/length ratio; H/W, height/width ratio; na, not available; W/L, width/length ratio. [file peerj-08-8652-s005.docx]

**Table S.2:**

**Selected vertebral measurements from PMO 224.248 in millimetres.**

Abbreviations: aa, atlas-axis complex; H/L, height/length ratio; H/W, height/width ratio; na, not available; W/L, width/length ratio.

| **Vertebral measurements of PMO 224.248** | | | | | | |
| --- | --- | --- | --- | --- | --- | --- |
| **Number** | **Height** | **Width** | **Length** | **H/L ratio** | **H/W ratio** | **W/L ratio** |
| **aa** | 21.80 | 22.10 | 39.00 | 0.56 | 0.99 | 0.57 |
| **3** | 21.00 | 27.40 | 24.70 | 0.85 | 0.77 | 1.11 |
| **4** | 22.50 | 28.40 | 25.60 | 0.88 | 0.79 | 1.11 |
| **5** | 22.40 | 29.00 | 27.70 | 0.81 | 0.77 | 1.05 |
| **6** | 23.10 | 30.40 | 27.60 | 0.84 | 0.76 | 1.10 |
| **7** | 23.40 | 31.60 | 30.00 | 0.78 | 0.74 | 1.05 |
| **8** | 25.00 | 31.90 | 30.40 | 0.82 | 0.78 | 1.05 |
| **9** | 24.80 | 32.60 | 32.10 | 0.77 | 0.76 | 1.02 |
| **10** | 25.50 | 34.50 | 32.70 | 0.78 | 0.74 | 1.06 |
| **11** | 26.00 | 35.80 | 35.10 | 0.74 | 0.73 | 1.02 |
| **12** | 26.10 | 35.40 | 36.00 | 0.73 | 0.74 | 0.98 |
| **13** | 26.90 | 37.80 | 38.10 | 0.71 | 0.71 | 0.99 |
| **14** | 28.50 | 38.70 | 38.30 | 0.74 | 0.74 | 1.01 |
| **15** | 29.20 | 38.90 | 39.90 | 0.73 | 0.75 | 0.97 |
| **16** | 30.00 | 41.40 | 41.20 | 0.73 | 0.72 | 1.00 |
| **17** | 30.90 | 43.00 | 42.30 | 0.73 | 0.72 | 1.02 |
| **18** | 31.70 | 43.20 | 43.10 | 0.74 | 0.73 | 1.00 |
| **19** | 33.00 | 44.00 | 43.90 | 0.75 | 0.75 | 1.00 |
| **20** | 34.50 | 45.50 | 44.20 | 0.78 | 0.76 | 1.03 |
| **21** | 35.60 | 48.17 | 45.20 | 0.79 | 0.74 | 1.07 |
| **22** | 35.00 | 48.40 | 45.50 | 0.77 | 0.72 | 1.06 |
| **23** | 35.90 | 49.70 | 44.00 | 0.82 | 0.72 | 1.13 |
| **24** | 36.00 | 53.00 | 46.00 | 0.78 | 0.68 | 1.15 |
| **25** | 37.50 | 51.00 | 48.00 | 0.78 | 0.74 | 1.06 |
| **26** | 39.00 | 53.00 | 47.00 | 0.83 | 0.74 | 1.13 |
| **27** | 39.00 | 54.00 | 50.00 | 0.78 | 0.72 | 1.08 |
| **28** | 39.00 | 54.00 | 51.00 | 0.76 | 0.72 | 1.06 |
| **29** | 40.00 | 56.50 | 51.00 | 0.78 | 0.71 | 1.11 |
| **30** | 40.60 | 57.70 | 52.70 | 0.77 | 0.70 | 1.09 |
| **31** | 39.50 | 59.00 | 54.00 | 0.73 | 0.67 | 1.09 |
| **32** | 42.20 | 60.00 | 53.20 | 0.79 | 0.70 | 1.13 |
| **33** | 43.40 | 61.80 | 53.00 | 0.82 | 0.70 | 1.17 |
| **34** | 42.40 | 62.00 | 53.00 | 0.80 | 0.68 | 1.17 |
| **35** | 43.40 | 61.30 | 52.90 | 0.82 | 0.71 | 1.16 |
| **36** | 43.20 | 64.20 | 52.30 | 0.83 | 0.67 | 1.23 |
| **37** | 44.90 | 64.90 | 55.10 | 0.81 | 0.69 | 1.18 |
| **38** | 42.20 | 65.40 | 54.80 | 0.77 | 0.65 | 1.19 |
| **39** | 48.40 | 63.90 | 54.90 | 0.88 | 0.76 | 1.16 |
| **40** | 46.30 | 66.40 | 55.40 | 0.84 | 0.70 | 1.20 |
| **41** | 48.20 | 67.90 | 56.30 | 0.86 | 0.71 | 1.21 |
| **42** | 50.10 | 69.80 | 57.10 | 0.88 | 0.72 | 1.22 |
| **43** | 47.50 | 72.20 | 54.70 | 0.87 | 0.66 | 1.32 |
| **44** | 49.40 | 71.30 | 53.60 | 0.92 | 0.69 | 1.33 |
| **45** | 46.70 | 70.10 | 53.50 | 0.87 | 0.67 | 1.31 |
| **46** | 45.90 | 71.00 | 52.10 | 0.88 | 0.65 | 1.36 |
| **47** | 46.70 | 74.30 | 54.50 | 0.86 | 0.63 | 1.36 |
| **48** | 52.50 | 72.30 | na | na | na | na |
| **49** | na | na | na | na | na | na |
| **50** | na | na | na | na | na | na |
| **51** | 52.90 | 78.50 | 49.5 | na | na | na |
| **52** | 52.60 | 76.40 | 57.9 | na | na | na |
| **53** | na | na | na | na | na | na |
| **54** | 52.1 | 73.9 | 58.1 | na | na | na |
| **55** | na | 76.6 | 59.2 | na | na | na |
| **56** | 54.4 | 72.2 | 59.2 | na | na | na |
| **57** | 55 | 71 | 55.5 | na | na | na |
| **58** | 57.5 | 71.1 | na | na | na | na |
| **59** | 59 | 68 | na | na | na | na |
