# Supplementary material for: A new plesiosaurian from the Jurassic–Cretaceous transitional interval of the Slottsmøya Member (Volgian), with insights into the cranial anatomy of cryptoclidids using computed tomography
Source: PeerJ. 2020 Mar 31;8:e8652. doi: 10.7717/peerj.8652 (PMC7120097; doi:10.7717/peerj.8652)
Supplement: Supplemental Information 6 [file peerj-08-8652-s006.docx]

**Table S.3:**

**Selected measurements from the pectoral girdle of PMO 224.248 in millimetres.**

| **Pectoral elements** | **mm** |
| --- | --- |
| **Interclavicle** |  |
| Preserved mediolateral width | ~100 |
| **Scapulae** |  |
| Total mediolateral width | 310 |
| **Right scapula** |  |
| Anteroposterior length | 190 |
| Mediolateral width | 155 |
| Length of glenoid facet | 50 |
| Dorsoventral height of glenoid facet | 40 |
| Length of coracoid facet | 50 |
| **Right coracoid** |  |
| Anteroposterior length (preserved) | 375 |
| Mediolateral width (at medial symphsis) | 175 |
| Dorsoventral height of medial symphsis | 60 |
| Anteriorposterior length anteromedial process | 70 |
| Mediolateral width anteromedial process (max) | 51 |
| Length of glenoid facet | 72 |
| Dorsoventral height of glenoid facet | 51 |
| Length of scapular facet | ~40 |
| Dorsoventral height of scapular facet | 51 |
