# Supplementary material for: A new plesiosaurian from the Jurassic–Cretaceous transitional interval of the Slottsmøya Member (Volgian), with insights into the cranial anatomy of cryptoclidids using computed tomography
Source: PeerJ. 2020 Mar 31;8:e8652. doi: 10.7717/peerj.8652 (PMC7120097; doi:10.7717/peerj.8652)
Supplement: Supplemental Information 7 — Abbreviations: pao, postaxial ossicle. [file peerj-08-8652-s007.docx]

**Table S.4:**

**Selected forelimb measurements from PMO 224.248 in millimetres.**

Abbreviations: pao, postaxial ossicle.

| **Left Forelimb** | **mm** | **Right Forelimb** | **mm** |
| --- | --- | --- | --- |
| **Left humerus** |  | **Right humerus** |  |
| Proximodistal length | 310 | Proximodistal length | 335 |
| Anteroposteriorwidth proximal end | 70 | Anteroposteriorwidth proximal end | 75 |
| Anteroposteriorwidth midshaft | 70 | Anteroposteriorwidth midshaft | 66 |
| Anteroposterior width distal end | 150 | Anteroposterior width distal end | 150 |
| Anteroposterior width radial facet | 60 | Anteroposterior width radial facet | 60 |
| Anteroposterior width ulnar facet | 70 | Anteroposterior width ulnar facet | 55 |
| Anteroposterior width pao facet | 72 | Anteroposterior width pao facet | 70 |
| Dorsoventral height (max) distal end | 39 | Dorsoventral height (max) distal end | 45 |
| **Left radius** |  | **Right radius** |  |
| Proximodistal length | 54 | Proximodistal length | 57 |
| Anteroposterior width | 67 | Anteroposterior width | 70 |
| Dorsoventral height (max) | 34 | Dorsoventral height (max) | 34 |
| **Left ulna** |  | **Right ulna** |  |
| Proximodistal length | 42 | Proximodistal length | 45 |
| Anteroposterior width | 60 | Anteroposterior width | 62 |
| Dorsoventral height (max) | 37 | Dorsoventral height (max) | 36 |
| **Left postaxial ossicle** |  | **Right postaxial ossicle** |  |
| Proximodistal length | 36 | Proximodistal length | 40 |
| Anteroposterior width | 35 | Anteroposterior width | 35 |
| Dorsoventral height (max) | 21 | Dorsoventral height (max) | 21 |
|  |  | **Right preaxial ossicle** |  |
|  |  | Proximodistal length | 19 |
|  |  | Anteroposterior width | 17 |
