# Supplementary material for: A new plesiosaurian from the Jurassic–Cretaceous transitional interval of the Slottsmøya Member (Volgian), with insights into the cranial anatomy of cryptoclidids using computed tomography
Source: PeerJ. 2020 Mar 31;8:e8652. doi: 10.7717/peerj.8652 (PMC7120097; doi:10.7717/peerj.8652)
Supplement: Supplemental Information 8 — Data compiled from specimen observation and Foffa (2018). [file peerj-08-8652-s008.docx]

**Table S.5:**

**Mechanical advantage measurements and calculations for selected cryptoclidid specimens.**

Data compiled from specimen observation and Foffa (2018).

| Specimen no | Genus | AMA outlever (mm) | AMA inlever (mm) | AMA (inlver/outlever) | PMA outlever (mm) | PMA inlever (mm) | PMA (inlver/outlever) |
| --- | --- | --- | --- | --- | --- | --- | --- |
| PMO 224.248* | *Ophthalmothule* | 210 | 28 | 0.13 | 64 | 28 | 0.44 |
| LEICS G18.19996.15 | *Muraneosaurus cf.* | 240 | 35 | 0.15 | 78 | 54 | 0.69 |
| PETMG R283 | *Cryptoclidus* | 270 | 51 | 0.19 | 74 | 54 | 0.73 |
| NHMUK R8431* | *Kimmerosaurus* | 240 | 30 | 0.13 | 59 | 30 | 0.51 |
| NHMUK R3539 | *Tricleidus* | 260 | 48 | 0.18 | 67 | 48 | 0.72 |
| SVB 1450* | *Spitrasaurus* | 220 | 24 | 0.11 | 77 | 24 | 0.31 |
| NHMUK R2421 | *Muraenosaurus* | 305 | 57 | 0.19 | 100 | 57 | 0.57 |
| NHMUK R2422 | *Muraenosaurus* | 290 | 58 | 0.20 | 90 | 58 | 0.64 |
| NHMUK PV R2860 | *Cryptoclidus* | 260 | 47 | 0.18 | 70 | 47 | 0.67 |
