# Supplementary figures and images for: A new plesiosaurian from the Jurassic–Cretaceous transitional interval of the Slottsmøya Member (Volgian), with insights into the cranial anatomy of cryptoclidids using computed tomography
Source: PeerJ. 2020 Mar 31;8:e8652. doi: 10.7717/peerj.8652 (PMC7120097; doi:10.7717/peerj.8652)

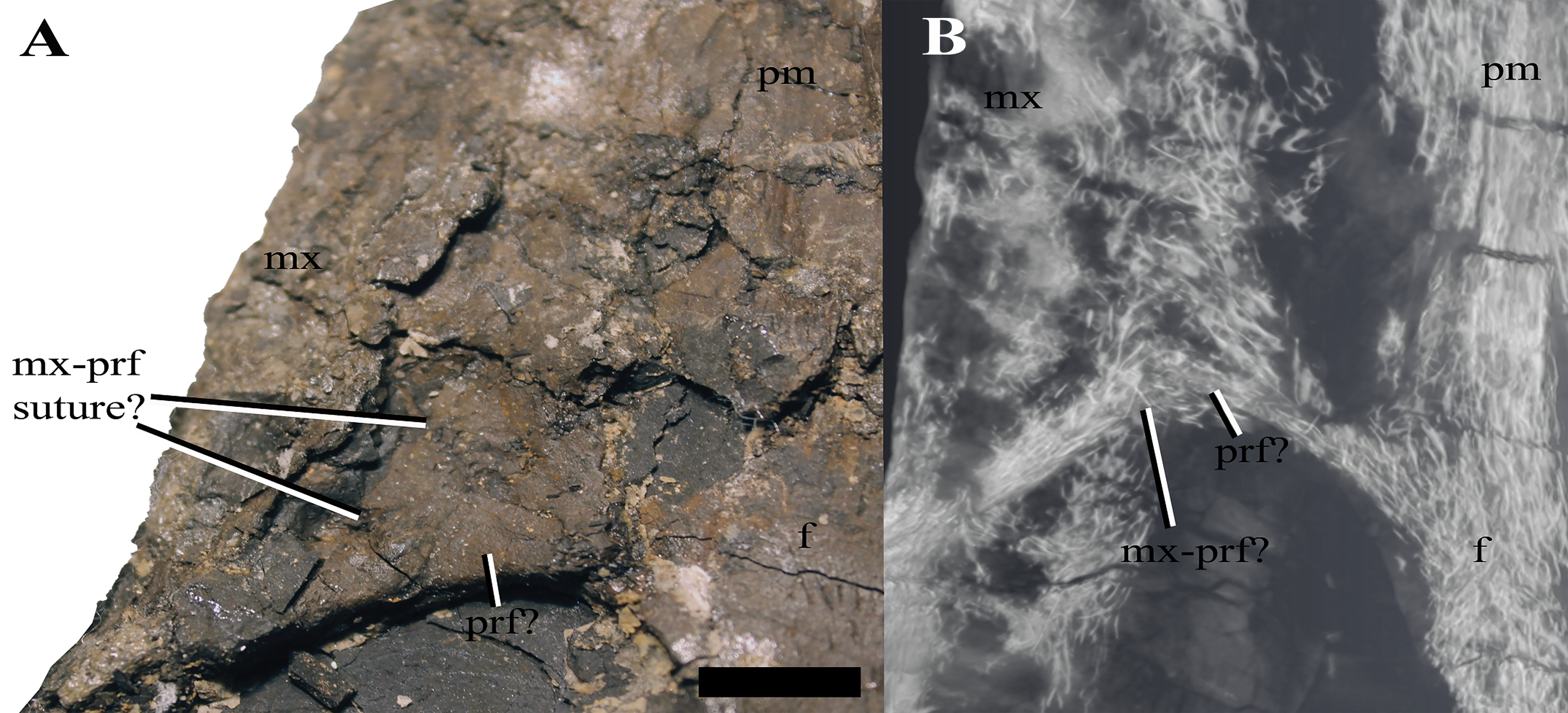

Supplement: Supplemental Information 9 — (A) photo of the left preorbital region, (B), a CT slice of the same area. Abbreviations: f, frontal; mx, maxilla; pm, premaxilla; prf, prefrontal. Scale bar equals 2 cm. Photograph by Aubrey Jane Roberts [file peerj-08-8652-s009.png]

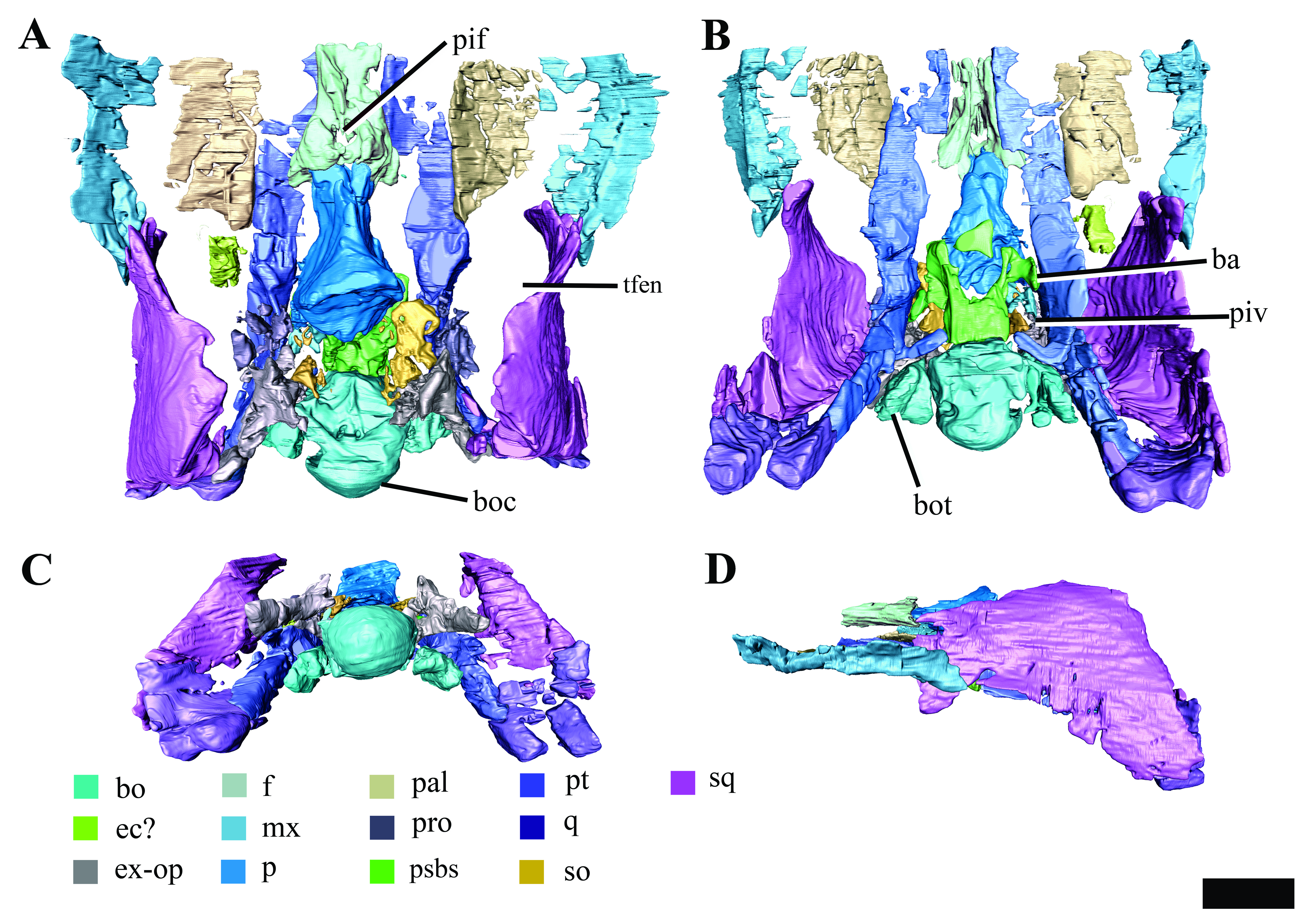

Supplement: Supplemental Information 10 — In (A) dorsal, (B) ventral, (C) posterior and (D) lateral views. Abbreveations: ba, basal articulation; bo, basioccipital; boc, basioccipital condyle; bot, basioccipital tuber; ec, ectopterygoid?; ex-op, exoccipital opisthotic; f, frontal; max, maxilla; p, parietal; pif, pineal foramen; piv, posterior interpterygoid vacuity; pro, prootic; psbs, parabasisphenoid; pt, pterygoid; q, quadrate; so, supraoccipital; sq, squamosal; tfen, temporal fenestra. Scale bar equals 2 cm. [file peerj-08-8652-s010.jpg]

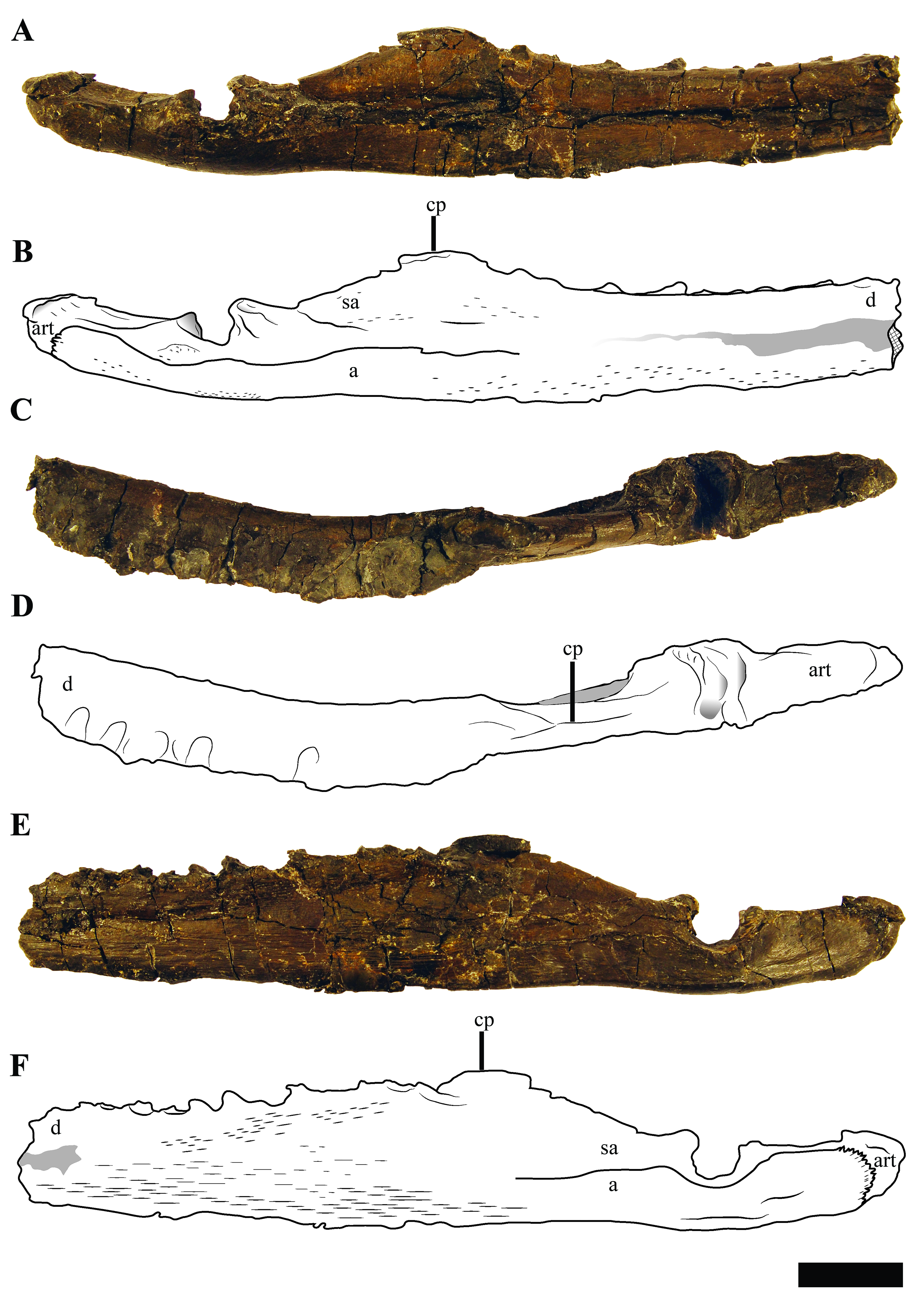

Supplement: Supplemental Information 11 — Photographs and interpretations in medial (A and B), dorsal (C and D) and lateral views (E and F). Abbreviations: a, angular; art, articular; cp, coronoid process; sa, surangular. Scale bar equals 2 cm. Photography by Aubrey Jane Roberts. [file peerj-08-8652-s011.jpg]

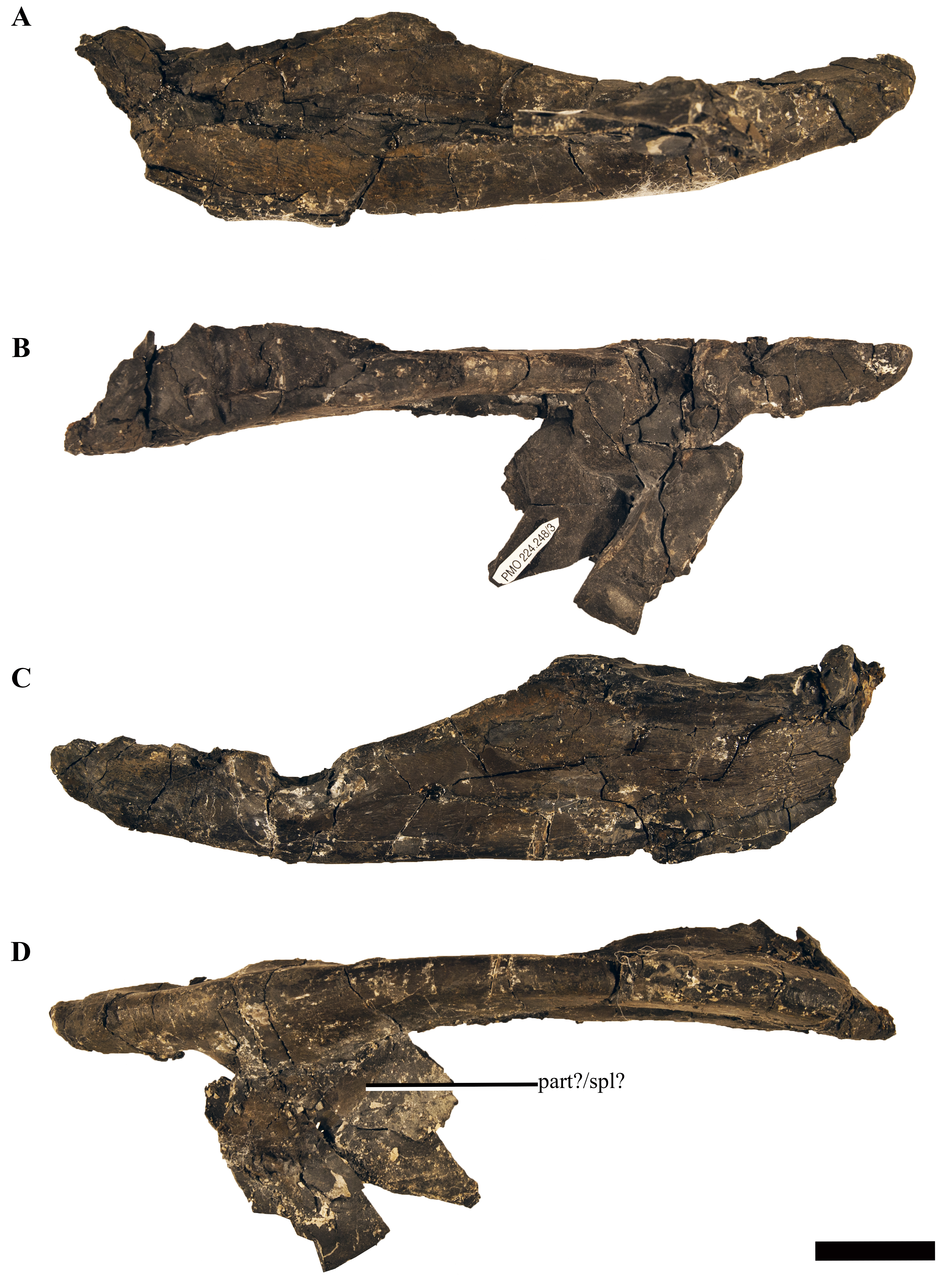

Supplement: Supplemental Information 12 — In (A) medial, (B) dorsal, (C) lateral and (D) ventral views. Abbreviations: part, prearticular; spl, splenial. Scale bar equals 2 cm. Photography by Aubrey Jane Roberts. [file peerj-08-8652-s012.jpg]

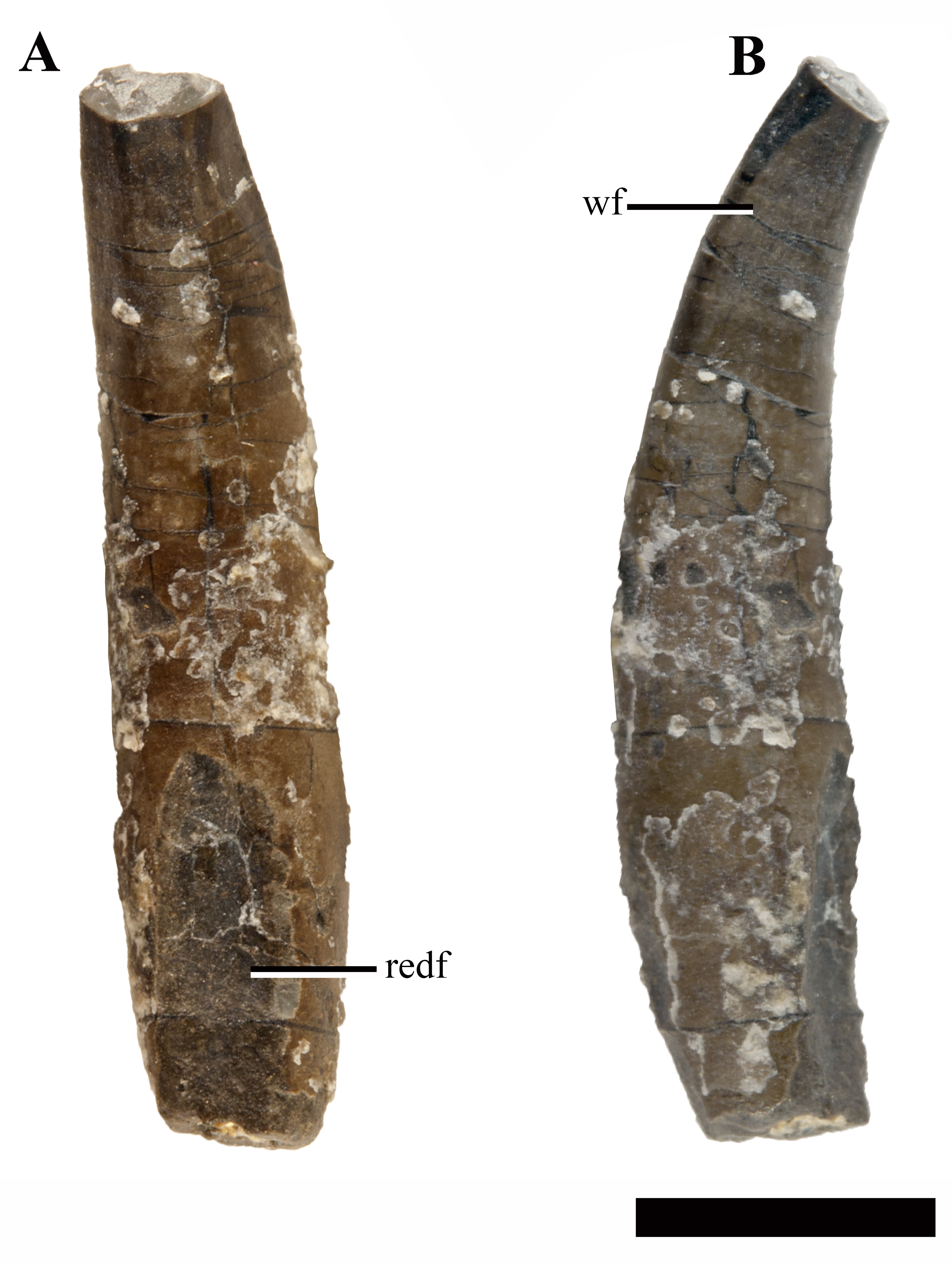

Supplement: Supplemental Information 13 — In (A) lingual and (B) axial views. Abbreviations: redf, reabsorption facet; wf, wear facet? Scale bar equals 0.5 cm. Photograph by Aubrey Jane Roberts. [file peerj-08-8652-s013.png]

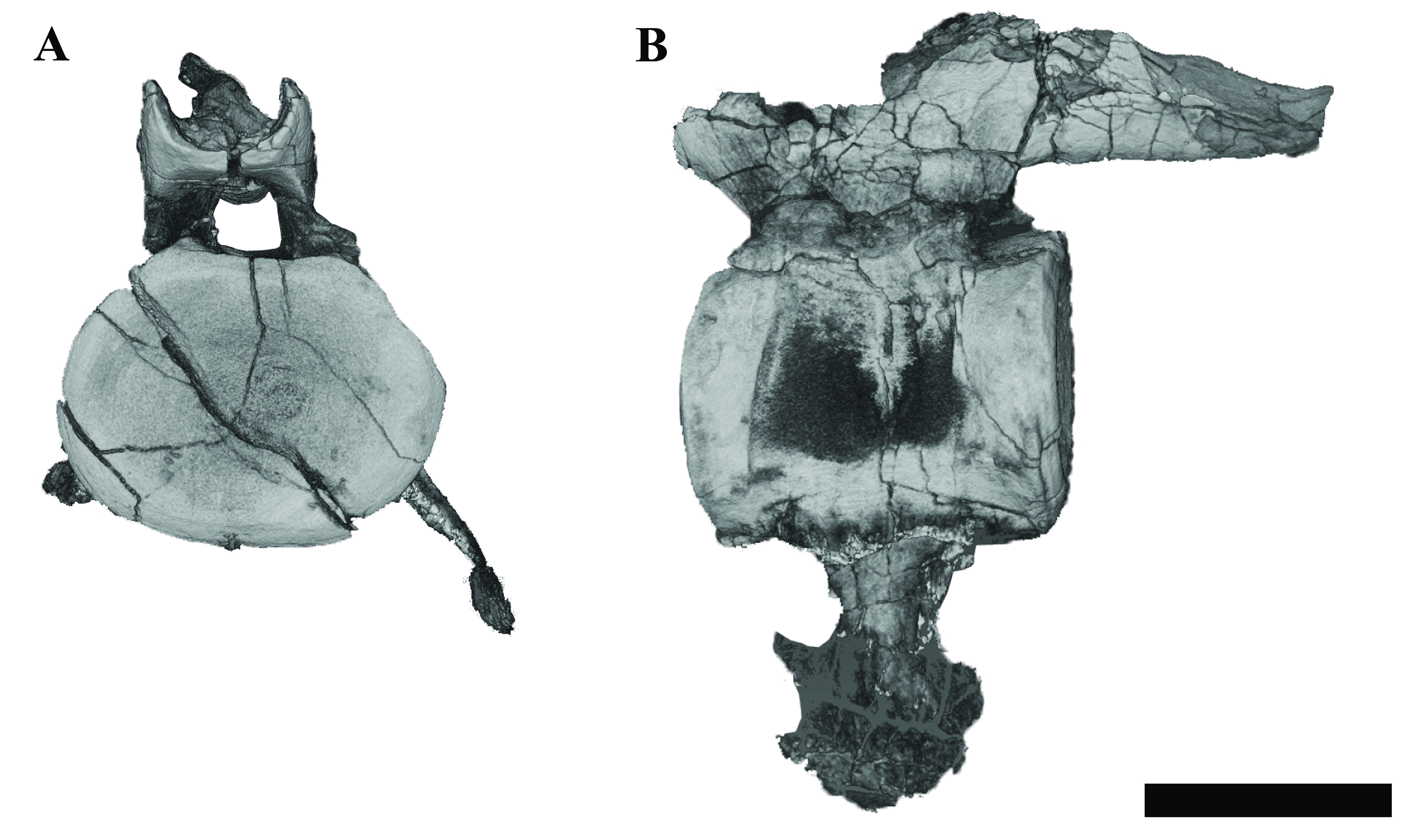

Supplement: Supplemental Information 14 — In A, anterior and B, lateral views. Scale bar equals 2 cm. [file peerj-08-8652-s014.jpg]

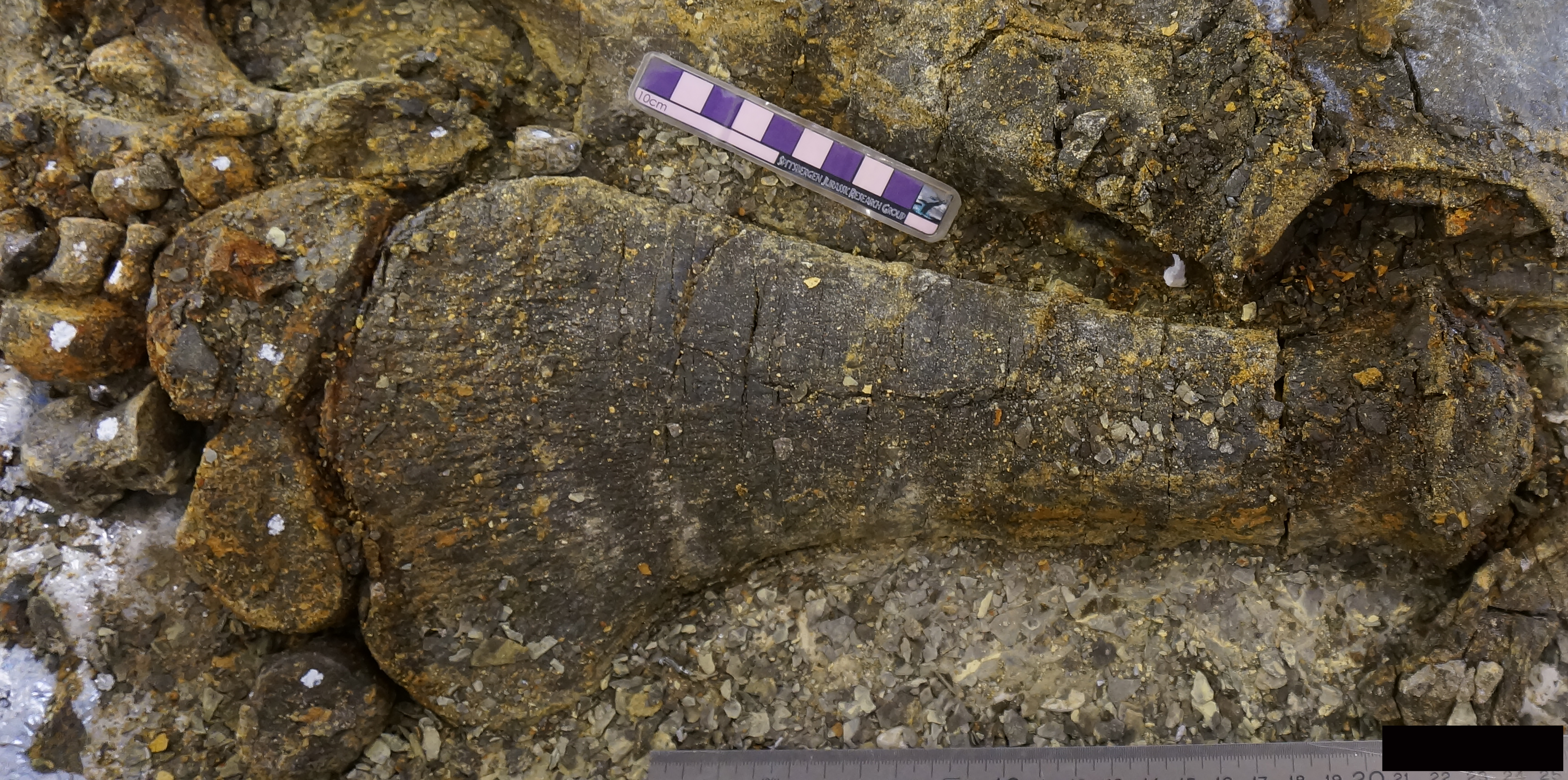

Supplement: Supplemental Information 15 — Scale bar equals 5 cm. Photograph by Aubrey Jane Roberts. [file peerj-08-8652-s015.png]

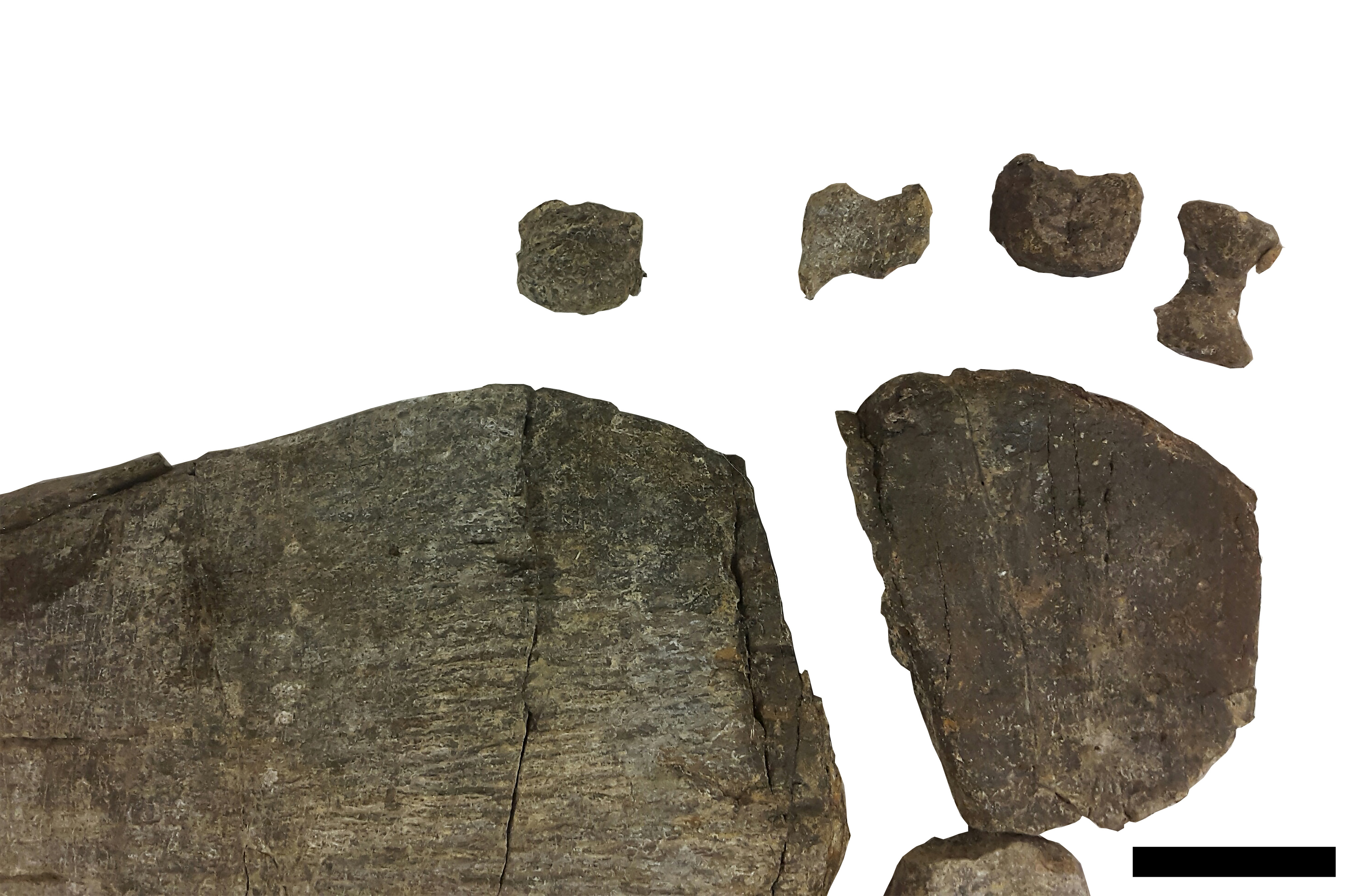

Supplement: Supplemental Information 16 — Scale equals 3 cm. Photograph by Lene Liebe Delsett. [file peerj-08-8652-s016.jpg]

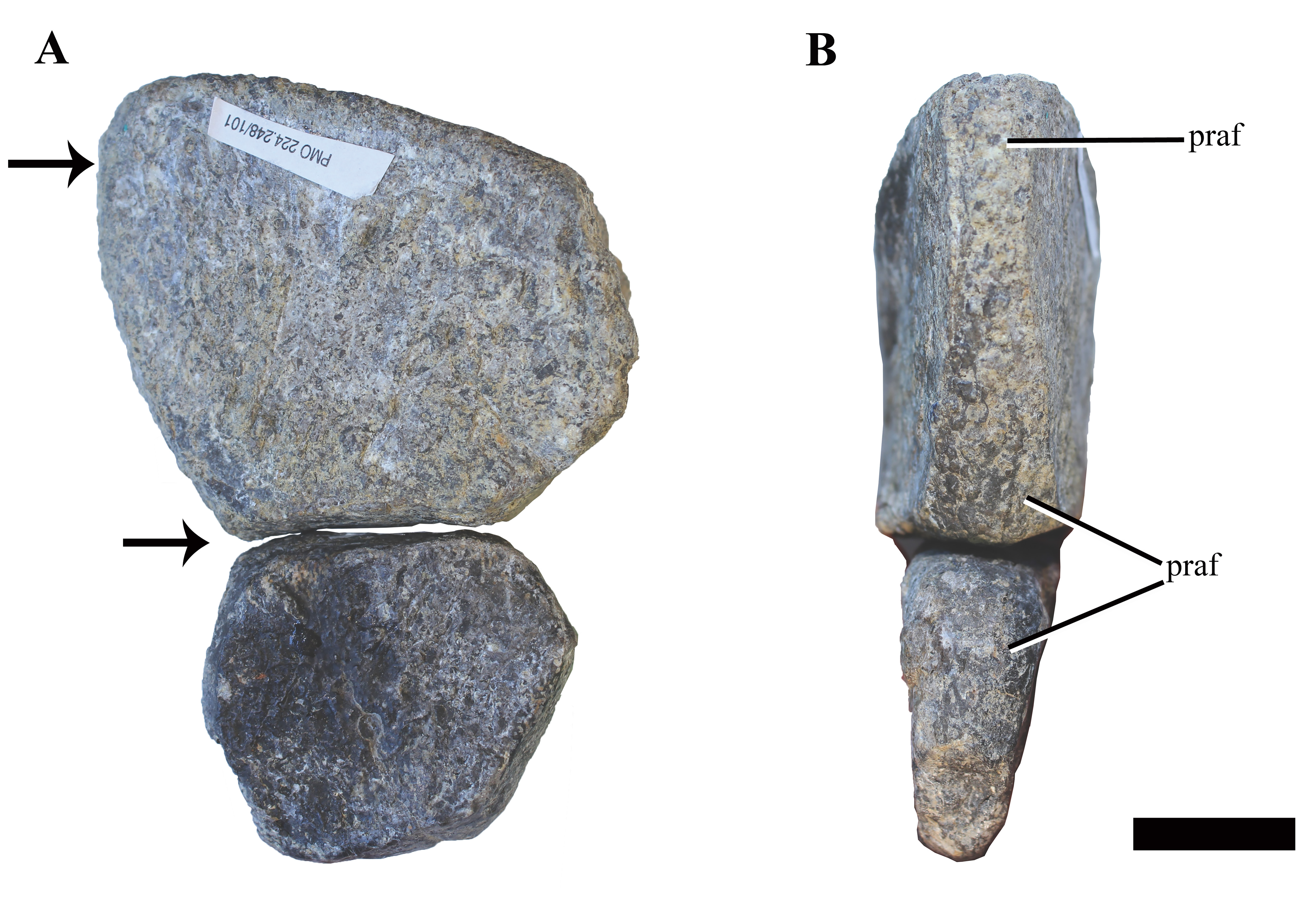

Supplement: Supplemental Information 17 — In (A) dorsal view and (B) anterior view. Arrows mark the point of possible insertion. Abbreviations: praf, preaxial facet. Scale equals 2 cm. Photography by Aubrey Jane Roberts. [file peerj-08-8652-s017.png]

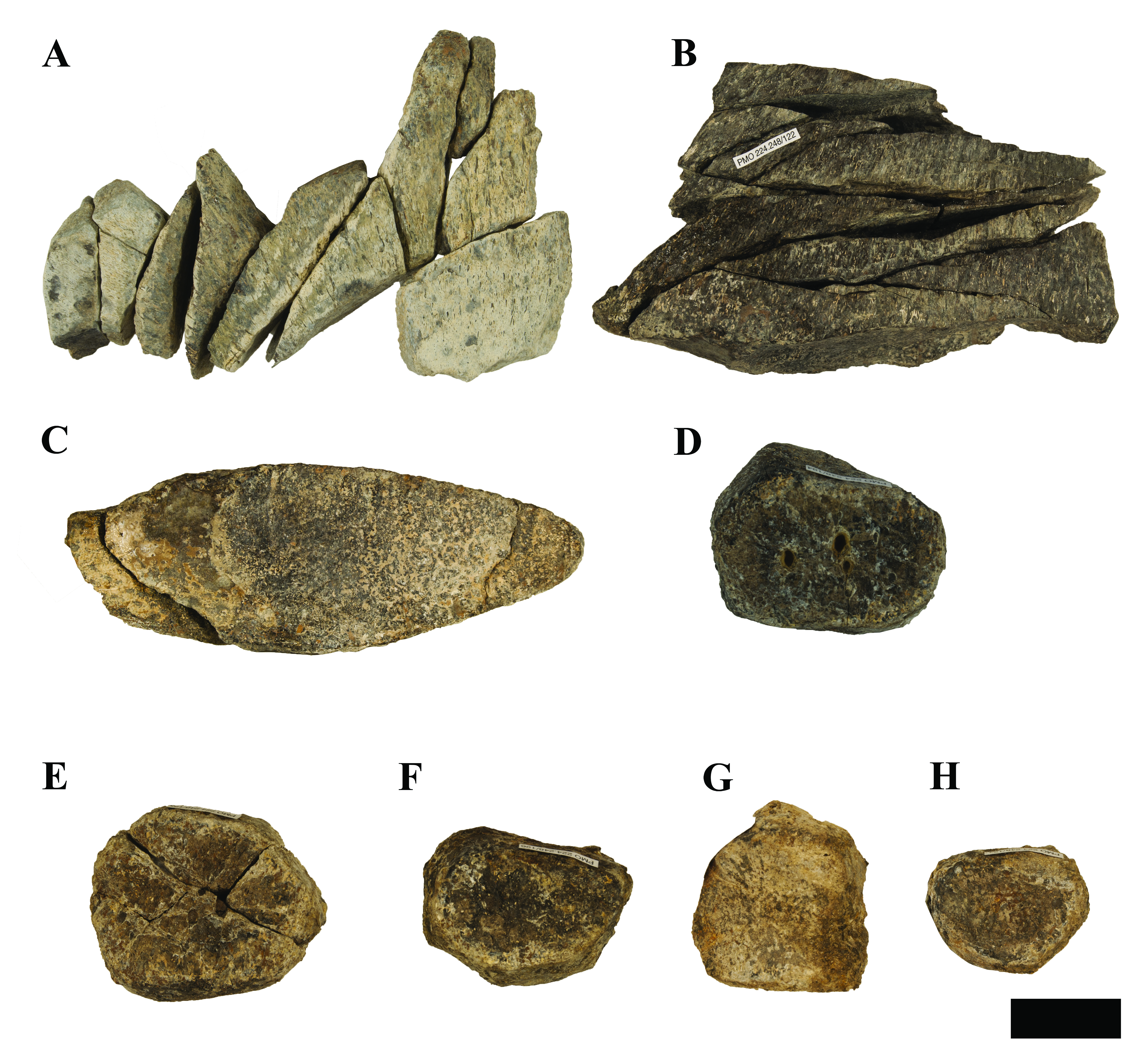

Supplement: Supplemental Information 18 — (A) the preserved distal portion of the right femur in dorsal view. The left femur in (B) ventral and (C) distal views. Mesopodial elements from the left limb: (D) astragalus; (E), fibulare; (F–H), distal tarsal elements. Scale equals 2 cm. Photography by Aubrey Jane Roberts. [file peerj-08-8652-s018.jpg]

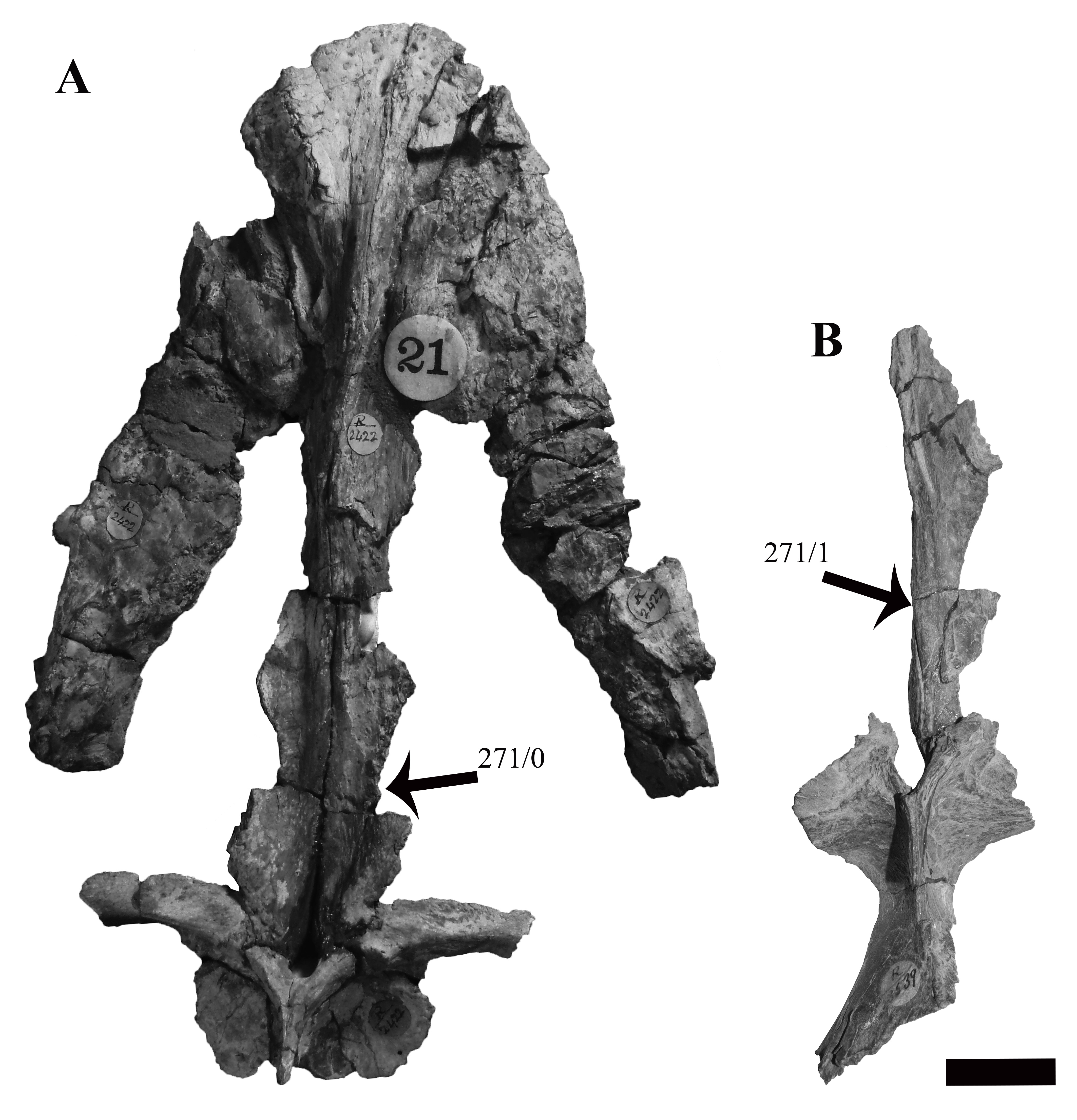

Supplement: Supplemental Information 19 — Arrows pointing to the areas of interest. (A) State 0 illustrated by Muraenosaurus leedsii NHMUK R2422 in dorsal view. Photo courtesy of R. Benson. (B) State 1 illustrated by Tricleidus seeleyi NHMUK R3539, in dorsal view. Scale bar equals 2 cm. Photography by Aubrey Jane Roberts. [file peerj-08-8652-s019.jpg]

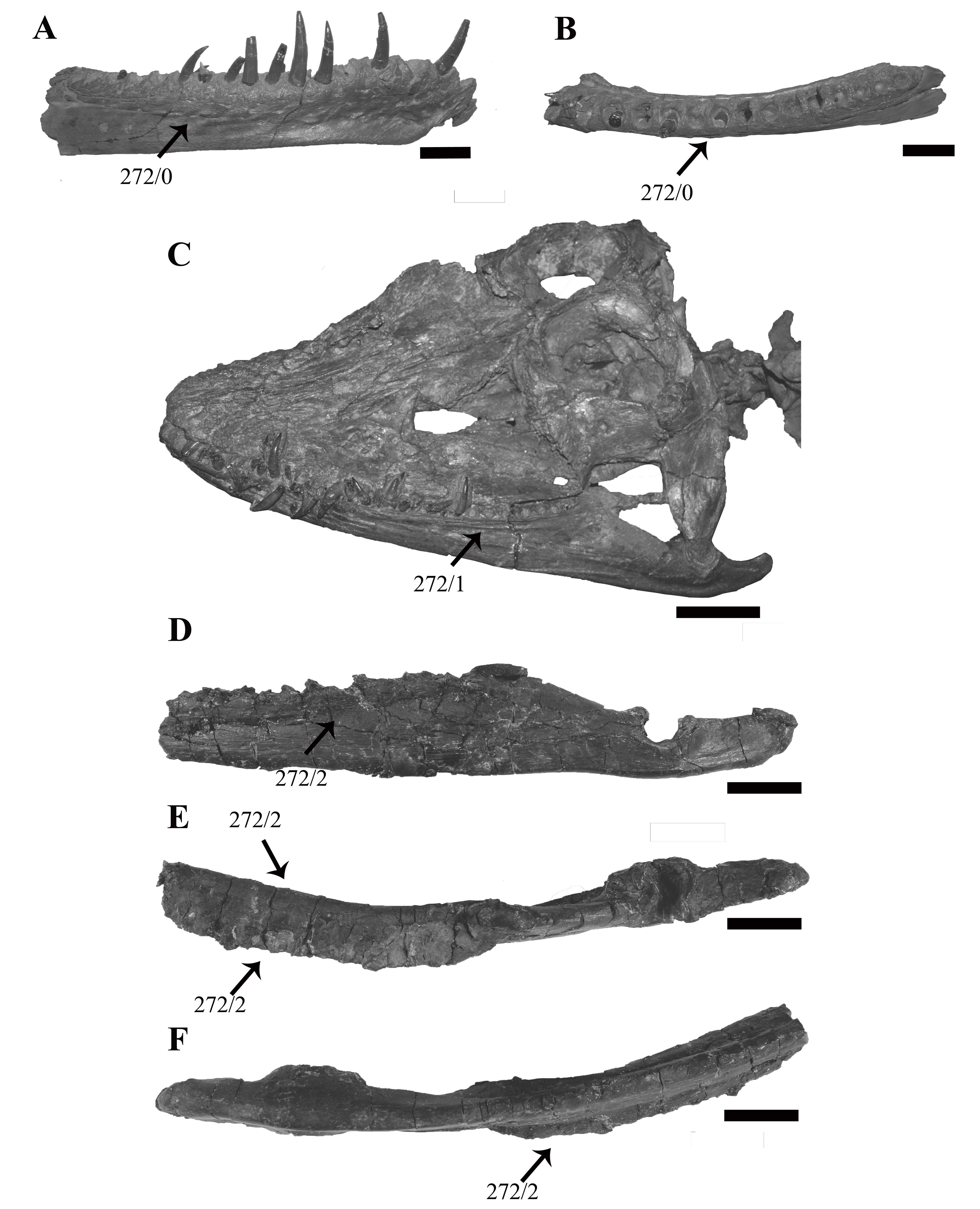

Supplement: Supplemental Information 20 — Arrows pointing to the areas of interest. State 272/0 illustrated by Tricleidus seeleyi NHMUK R3539 in (A) lateral and (B) dorsal views. State 272/1 illustrated by Cryptoclidus eurymerus PETMG R.283.412 in (C) lateral view. State 272/2 illustrated by PMO 224.248 in (D) lateral, (E) dorsal and (F) ventral views. Scale bars equal 2 cm in (A and B), (D–F). Scale bar equals 4 cm in (C). Photography by Aubrey Jane Roberts. [file peerj-08-8652-s020.jpg]

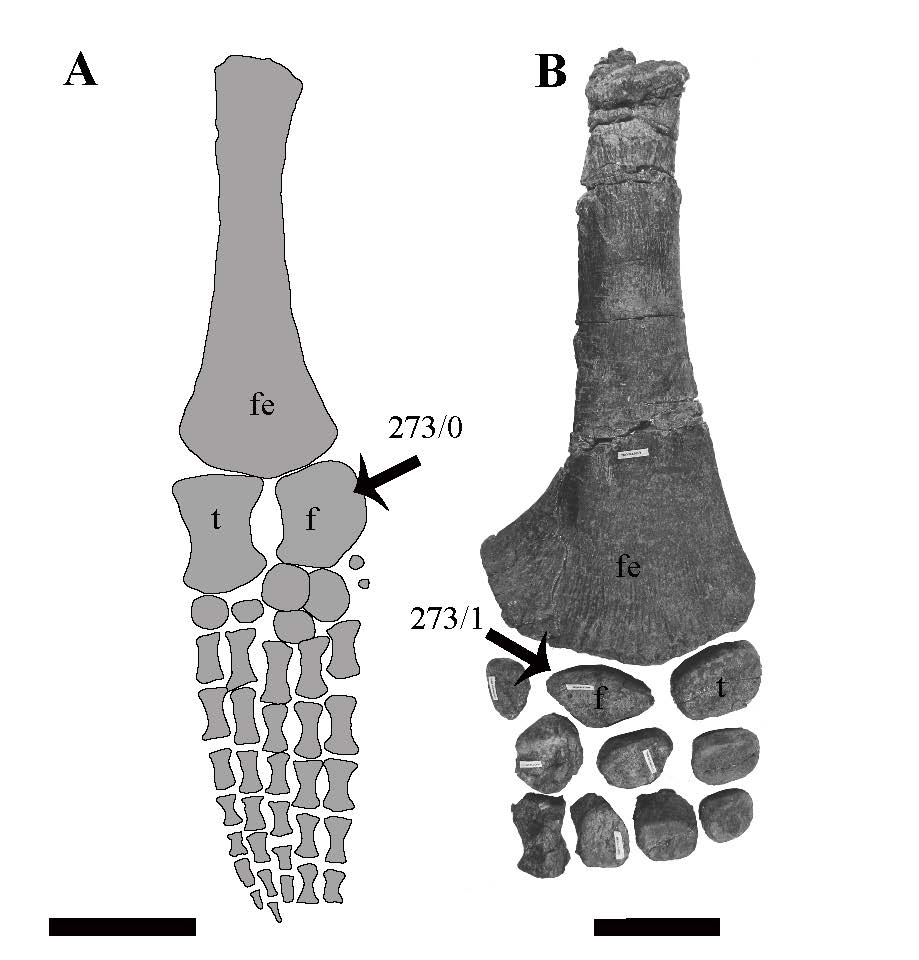

Supplement: Supplemental Information 21 — Arrows pointing to the areas of interest. (A) State 0 illustrated by Hauffiosaurus zanoi modified from a line drawing based of Vincent (2011) in dorsal view. (B) State 1 illustrated by Colymbosaurus svalbardensis modified from Roberts et al. (2017) in dorsal view. Abbreviations: f, fibula; fe, femur; t, tibia. Scale bars equal 10 cm. Photography by Aubrey Jane Roberts. [file peerj-08-8652-s021.jpg]
